# Development and Pilot Validation of the Romanian Antioxidant Cosmetics and Nutrition Questionnaire (RO-ACNQ): A Simulation-Based Multicenter Benchmark with an Observed Pilot Sample

**DOI:** 10.3390/antiox15101282

**Published:** 2026-10-03

**Authors:** Radu Dumitru Moleriu, Teodora Piroș, Ruxandra-Cristina Marin, Călin Muntean, Lavinia Cristina Moleriu, Adrian-Cosmin Ilie

**Affiliations:** 1 Departament III Functional Science, Discipline of Medical Informatics and Biostatistics, “Victor Babeș” University of Medicine and Pharmacy, 300041 Timisoara, Romania; radu.moleriu@umft.ro (R.D.M.); teodora.piros@student.umft.ro (T.P.); moleriu.lavinia@umft.ro (L.C.M.); 2 Discipline of Pharmacology, Clinical Pharmacology and Pharmacotherapy, “Carol Davila” University of Medicine and Pharmacy, 050474 Bucharest, Romania; 3 Doctoral School of Biological and Biomedical Sciences, University of Oradea, 410087 Oradea, Romania; 4 Departament III Functional Science, Discipline of Public Health and Health Management, “Victor Babeș” University of Medicine and Pharmacy, 300041 Timisoara, Romania, 300041 Timisoara, Romania; ilie.adrian@umft.ro

**Keywords:** antioxidant cosmetics, oxidative stress, questionnaire validation, psychometrics, exploratory factor analysis, confirmatory factor analysis, structural equation modeling, nutrition, skin health, university students

## Abstract

Oxidative stress contributes substantially to skin aging, while antioxidant-based cosmetics and nutrition-oriented “skin-from-within” strategies are increasingly popular among young adults. However, no validated Romanian instrument integrates antioxidant-related knowledge, attitudes, nutrition/lifestyle behaviors, and cosmetic-use intentions. This study developed the 61-item Romanian Antioxidant Cosmetics and Nutrition Questionnaire (RO-ACNQ), including a 36-item psychometric core covering four hypothesized domains: Knowledge, Attitudes, Behavioral Intention, and Nutrition/Lifestyle. Initial psychometric evaluation used a unified dataset (*N* = 1013) comprising a reproducible synthetic multicenter benchmark (*n* = 957) and an observed pilot sample (*n* = 56). Exploratory factor analysis (KMO = 0.943) identified eight weak or structurally inconsistent items, yielding a refined 28-item, four-factor model with acceptable-to-good internal consistency (α = 0.70–0.89) and good fit in an internal, same-sample CFA evaluation (χ^2^/df = 1.43, CFI = 0.984, TLI = 0.982, RMSEA = 0.021, SRMR = 0.033). Discriminant validity was generally supported (HTMT ≤ 0.74), although convergent validity remained partial. Within the simulation-based benchmark, theoretically expected known-groups patterns, high synthetic test–retest stability (ICC ≥ 0.97), and a Knowledge → Attitudes → Intention pathway were reproduced. Importantly, the refined domains showed encouraging internal consistency in the observed participants (α = 0.78–0.89; total α = 0.91), while differences between observed and synthetic response distributions identified areas requiring further calibration. These findings support the RO-ACNQ as a promising candidate instrument and establish a refined 28-item structure for prospective multicenter validation in participant-derived data.

## 1. Introduction

Oxidative stress, defined as an imbalance between the generation of reactive oxygen species (ROS) and the capacity of enzymatic and non-enzymatic antioxidant defenses, is a major mechanism contributing to both intrinsic (chronological) and extrinsic skin aging [1,2]. In the skin, excessive ROS production promotes oxidative damage to cellular lipids, proteins, and nucleic acids and activates inflammatory and matrix-degrading pathways, contributing to collagen degradation, extracellular-matrix remodeling, epidermal-barrier dysfunction, and clinical manifestations such as wrinkling, dryness, uneven pigmentation, and loss of elasticity [3]. Recent evidence further places oxidative stress at the interface between intrinsic aging and the cumulative effects of the skin exposome, through which multiple environmental and lifestyle factors may converge on common redox-sensitive pathways. Ultraviolet radiation remains a major external driver, but visible and infrared radiation, environmental pollutants, cigarette smoke, psychological stress, and lifestyle- and diet-related factors may also increase cutaneous oxidative burden [2,3]. In particular, visible light is increasingly recognized as biologically active in the skin, where ROS generation may contribute to photoaging, extracellular-matrix degradation, and persistent pigmentation [3,4]. Accordingly, modulation of oxidative stress and reinforcement of cutaneous antioxidant defenses have become important targets in contemporary preventive dermatology and cosmetic science.

In response, both topical and nutritional antioxidant strategies have been extensively investigated. Topical agents (including L-ascorbic acid (vitamin C), tocopherol (vitamin E), niacinamide, ferulic and other phenolic acids, coenzyme Q10, and plant-derived polyphenols such as quercetin) can neutralize ROS, support repair processes, and provide complementary photoprotection when combined with conventional ultraviolet filters [5,6,7,8]. Recent evidence continues to emphasize the potential of naturally derived antioxidant compounds in targeting oxidative damage, inflammation, collagen degradation, and other mechanisms involved in cutaneous aging [9,10].

In parallel, “skin-from-within” approaches emphasize dietary patterns rich in antioxidant-containing foods, including fruits, vegetables, nuts, tea, and other sources of polyphenols, carotenoids, vitamins, and micronutrients, together with selected nutritional supplementation [11,12,13]. The diversity of naturally occurring dietary antioxidants is also evident in food matrices such as honey, where phenolic acids and flavonoids contribute substantially to antioxidant potential. Recent analysis of Romanian monofloral honeys identified marked botanical variation in phenolic composition and antioxidant capacity, illustrating that the antioxidant characteristics of natural foods depend not only on food category but also on their specific phytochemical profile and botanical origin [14]. This nutritional dimension is increasingly incorporated into the broader concept of nutritional dermatology, which recognizes interactions among dietary exposure, systemic and cutaneous oxidative stress, inflammatory pathways, extracellular-matrix integrity, epidermal function, and skin aging. Plant-derived antioxidants, including polyphenols, carotenoids, tocopherols, and ascorbic acid, have attracted particular interest because of their capacity to modulate oxidative and inflammatory processes implicated in cutaneous aging [15].

However, the biological plausibility of dietary antioxidant strategies should be distinguished from the strength of clinical evidence supporting specific interventions. A recent systematic review of antioxidant supplementation in dermatology found preliminary evidence for benefits of topical and oral antioxidants in outcomes including photodamage and photoaging, while also highlighting heterogeneity across compounds and applications. Recent meta-analyses similarly emphasize the potential contribution of dietary antioxidants and phytochemicals to skin health while noting substantial variation according to the compound, dose, bioavailability, formulation, duration of exposure, and clinical outcome evaluated. This distinction is particularly important from a consumer perspective because broad perceptions of antioxidant benefit may extend beyond the strength or specificity of the available evidence [13,16,17].

The convergence between nutrition and cosmetics is particularly relevant to antioxidant research. Consumers are increasingly exposed to antioxidants not only as ingredients in topical formulations but also through functional foods, supplements, and “beauty-from-within” concepts. Contemporary research has consequently begun to examine food-derived bioactive compounds as potential skincare ingredients and to consider topical and nutritional approaches within a more integrated framework. At the same time, evidence supporting individual interventions remains heterogeneous; even for oral products marketed for photoprotection or skin aging, efficacy differs considerably among antioxidant classes and clinical endpoints. This evolving evidence base makes the ability of consumers to distinguish general antioxidant claims from evidence-based applications increasingly relevant [18,19].

Consumer interest in “clean beauty,” natural antioxidants, and preventive skincare has surged among adolescents and young adults, driven to a large extent by social media exposure, influencer marketing, and curated beauty standards [20,21,22]. University students represent a particularly relevant population because they are intensively exposed to digital health and cosmetic information, frequently experiment with cosmetic products, and are at a life stage during which health-related behaviors and purchasing patterns are being established. However, frequent cosmetic use does not necessarily imply adequate knowledge. Recent evidence among university students has identified substantial knowledge gaps despite widespread cosmetic consumption, together with frequent self-reported cosmetic-related adverse effects [23]. Such findings reinforce the need to distinguish product use from informed, evidence-based use.

This distinction may be particularly important for antioxidant-containing products. Antioxidants are commonly associated with broad concepts such as “anti-aging,” “natural,” “protective,” or “healthy,” yet these perceptions may reflect markedly different levels of understanding regarding oxidative stress, active ingredients, dietary sources, expected benefits, limitations, and appropriate use. Moreover, favorable perceptions of natural or antioxidant-containing products should not be assumed to reflect evidence-based knowledge. Contemporary consumer research indicates that health- and environment-related product claims interact with consumer concerns and knowledge in shaping attitudes and behavioral intentions toward personal-care products [24]. Similarly, research on organic personal-care products has identified product knowledge, information adoption, environmental value, convenience, and perceived quality as relevant components of purchase decision-making [25]. Thus, knowledge alone may not translate directly into behavior; attitudes toward efficacy and safety, trust in products and information sources, professional recommendations, price, marketing exposure, and perceived personal relevance may influence whether antioxidant-related knowledge ultimately affects cosmetic choices or nutritional practices. An antioxidant-focused assessment should therefore capture not only what individuals know about antioxidants but also how this knowledge relates to their evaluations, lifestyle behaviors, and intentions.

Despite the growing scientific and consumer interest in antioxidants, to our knowledge no validated Romanian-language instrument currently provides an integrated assessment of antioxidant-related knowledge, attitudes, nutrition/lifestyle practices, cosmetic-use behaviors, and behavioral intention among university students. Established dermatological instruments—including the Dermatology Life Quality Index (DLQI), Skindex-29/Skindex-17, the Sensitive Scale-10, and the Impact of Chronic Skin Disease on Daily Life (ISDL)—primarily quantify symptoms, sensitivity, or quality-of-life burden associated with established skin conditions rather than preventive knowledge and behavior in young populations [26,27,28]. Conversely, conventional knowledge–attitude–practice or nutrition-literacy questionnaires generally do not integrate antioxidant-specific cosmetic and nutritional behaviors. This creates a measurement gap at the intersection of antioxidant science, nutritional dermatology, cosmetic behavior, and preventive health.

A culturally appropriate multidimensional instrument could address this gap by identifying discrepancies between antioxidant knowledge and actual practices, characterizing attitudes that may facilitate or discourage evidence-based product use, and distinguishing nutritional behaviors from cosmetic-use intentions. Such an instrument may also provide a standardized outcome for educational interventions and, after full validation, facilitate comparisons across academic backgrounds and other relevant student subgroups. Importantly, its purpose would not be to infer biological antioxidant status or clinical skin outcomes from self-report, but rather to characterize the cognitive and behavioral dimensions through which scientific information about antioxidants may be translated into everyday nutrition and skincare choices.

The present study therefore describes the development and initial psychometric evaluation of the Romanian Antioxidant Cosmetics and Nutrition Questionnaire (RO-ACNQ), a multidimensional instrument designed to assess antioxidant-related Knowledge, Attitudes toward antioxidant cosmetics, Behavioral Intention, and Nutrition/Lifestyle behaviors among university students, complemented by descriptive information on cosmetic use and perceived skin health. The study was designed as an instrument-development and simulation-based psychometric benchmarking phase augmented by an observed pilot sample, rather than as definitive empirical validation. The synthetic multicenter benchmark was used to stress-test the proposed measurement structure, scoring procedures, and prespecified analytical workflow, while the first participant-derived responses provided an initial empirical assessment of reliability and a means of identifying discrepancies between simulated and observed response distributions. Within this framework, the research examined whether the proposed domains showed adequate internal consistency and factorial differentiation, whether the refined measurement structure demonstrated acceptable convergent and discriminant properties, whether theoretically expected known-groups patterns could be reproduced, and whether Knowledge, Attitudes, and Behavioral Intention formed a coherent socio-cognitive pathway [29]. The overarching objective was to identify a psychometrically defensible candidate structure for subsequent prospective multicenter validation in real university populations, while explicitly distinguishing simulation-supported methodological evidence from participant-derived evidence.

## 2. Materials and Methods

### 2.1. Study Design and Data Sources

This methodological pilot study combined a simulation-based benchmark with an initial observed sample. The benchmark comprised 957 synthetic response profiles generated to evaluate the proposed RO-ACNQ measurement structure and psychometric analysis pipeline before full field validation. The observed pilot comprised 56 first-year Medicine and Pharmacy students who completed the questionnaire anonymously through Microsoft Forms and provided preliminary empirical calibration of the simulated framework. Synthetic and observed data were distinguished throughout the analyses and interpreted separately.

The synthetic benchmark was designed to emulate the planned multicenter validation study among Romanian university students from four institutions: the “Victor Babeș” University of Medicine and Pharmacy Timișoara (UMFT), West University of Timișoara (UVT), “Aurel Vlaicu” University of Arad (UAV), and “Carol Davila” University of Medicine and Pharmacy Bucharest (UMFCD). All questionnaire variables were simulated, not only the psychometric core. The synthetic profiles were generated according to the predefined simulation framework, with center allocation following the planned quotas (240/239/239/239). Age was drawn from a right-skewed truncated discrete distribution spanning 18–35 years, and sex, residence, study level, academic field, lifestyle descriptors (Section A), cosmetic-use variables (Section B), and perceived-skin-health variables (Section G) were sampled from prespecified categorical marginal distributions chosen to approximate the intended recruitment pool of the four centers: predominantly female and urban, mostly aged 19–24 years, with Medicine/Pharmacy records intentionally overrepresented (about one half) to enable known-groups testing. These targets are consistent with published data on Romanian and European student populations [30,31], and the realized synthetic marginal distributions are reported exhaustively in Table 2 and Supplementary Table S3. The 36 core Likert items (1–5) were generated from a latent-variable model: for each profile, four correlated domain-level latent traits (Knowledge, Attitudes, Intention, Nutrition/Lifestyle) with prespecified positive interdependencies, strongest between Attitudes and Intention and between Knowledge and Attitudes, were shifted according to academic profile, sex, and cosmetic-use status, and each item response was obtained by thresholding its domain trait plus item-specific noise into five ordinal categories. The realized benchmark also embeds a subset of weakly or inconsistently related items: five skeptical/marketing Attitudes items (Q45–Q47, Q49, Q50) carry essentially no shared variance with the Attitudes trait, two items (Q31, Q51) are driven primarily by the knowledge-related trait, and one item (Q27) is essentially independent of all four traits. No parameter of the generator was estimated or tuned using the observed pilot responses; the demographic targets relied only on published aggregate statistics, and the substantial distributional differences between synthetic and observed responses (Section 3.9) are consistent with this independence. A matched synthetic retest dataset of 200 profiles (maximum 50 per center) was additionally generated to benchmark the planned test–retest procedures. The retest profiles favored the 19–24-year range with a controlled sex distribution and reproduced baseline responses with moderate prespecified test–retest variation.

The two data sources were harmonized using a predefined variable dictionary, standardized response labels, and a source indicator identifying each record as synthetic or observed. Quality control yielded 1013 unique records (957 synthetic and 56 observed), with no duplicate identifiers.

Simulation-based analyses were considered methodological benchmarks rather than empirical validation evidence. Findings from the 56 observed participants are therefore reported as preliminary pilot results, while definitive psychometric validation of the RO-ACNQ will require completion of the ongoing prospective multicenter study. Because the generator imposes the four-domain structure and the inter-construct dependencies, recovery of that structure by the factor-analytic and structural models is expected by construction; such results are reported as verification of the analysis pipeline, not as evidence about the instrument. The informative simulation outcomes are of a different kind: whether the prespecified item-screening rules isolate exactly the weakly related items embedded in the generator, whether fit indices, reliability coefficients, and effect-size estimates behave as intended at the planned sample size, and whether the refined structure subsequently transfers to the observed participants.

### 2.2. Questionnaire Development and Structure

The Romanian Antioxidant Cosmetics and Nutrition Questionnaire (RO-ACNQ) was developed through a multistep process consistent with established recommendations for questionnaire and scale development [30,31,32]. The development process comprised: (1) a structured review of the literature addressing oxidative stress and skin health, topical and dietary antioxidants, antioxidant-related nutrition and lifestyle behaviors, cosmetic-use patterns, and relevant dermatological and behavioral instruments; (2) generation of an initial item pool by a multidisciplinary team with expertise in dermatology, nutrition, public health, psychology, and biostatistics; (3) expert review for content relevance, clarity, and coverage of the intended constructs; (4) cognitive pre-testing in a small group of university students to assess comprehensibility, cultural appropriateness, and completion time (approximately 12–15 min); and (5) iterative refinement of item wording and questionnaire structure.

The resulting RO-ACNQ comprises 61 numbered items organized into seven sections (A–G), combining 25 descriptive items with a 36-item psychometric core (Table 1). Each numbered question is counted as one item; the food-frequency matrix (Q18) is therefore counted as a single item comprising nine rated food rows, which are expanded into separate variables in the analytical database (69 harmonized variables in total). Sections A (10 items) and B (6 items) collect socio-demographic, lifestyle, and antioxidant-cosmetic-use characteristics; Section C additionally contains 5 descriptive dietary items (daily fruit and vegetable intake, the Q18 food-frequency matrix, antioxidant-supplement use and types, and the perceived influence of diet on skin health); and Section G (4 items) assesses perceived skin health and related factors. These 25 descriptive items are intended primarily for descriptive and exploratory analyses and are not included in the latent measurement model. Sections C–F comprise the 36-item psychometric core, organized into four a priori domains: Nutrition/Lifestyle (10 items), Knowledge of oxidative stress and antioxidants (10 items), Attitudes toward antioxidant cosmetics (10 items), and Behavioral Intention (6 items).

Items assessing Knowledge, Attitudes, and Behavioral Intention were rated on a five-point agreement scale ranging from 1 (*totally disagree*) to 5 (*totally agree*). Nutrition/Lifestyle items used a parallel five-point frequency scale ranging from 1 (*never*) to 5 (*very frequently*). Two negatively worded items, Q26 (frequent consumption of ultra-processed foods) and Q41 (“I do not consider myself sufficiently informed about antioxidants”), were reverse-scored before calculation of domain scores and psychometric analyses. Higher scores therefore consistently represented greater antioxidant-related knowledge, more favorable attitudes and behavioral intentions, and healthier antioxidant-oriented nutrition/lifestyle behaviors.

For psychometric evaluation, domain scores were calculated as the mean of the constituent items, provided that the required items were available. The original 36-item structure was treated as the a priori measurement model. Item retention and refinement were subsequently determined from the psychometric analyses described in Section 2.3 rather than specified in advance. Consequently, the refined structure reported in the Results represents an analysis-derived model and not a predetermined questionnaire configuration.

The complete Romanian-language questionnaire, together with its English translation, administration instructions, and scoring information, is provided in Supplementary File S1.

### 2.3. Statistical Analysis

Statistical analyses were performed using JASP (version 2.3; JASP Team, Amsterdam, The Netherlands), with two-tailed statistical significance set at α = 0.05. Analyses of the combined analytical dataset were interpreted as simulation-based methodological benchmarks because synthetic profiles constituted the majority of records, whereas analyses restricted to the observed pilot sample were considered preliminary empirical evaluations.

Distributional characteristics were assessed using the Shapiro–Wilk test, skewness, and kurtosis. As item and domain-score distributions departed from normality, continuous variables are summarized as medians and interquartile ranges (IQR), and non-parametric procedures were used for group comparisons. Item-level analyses included medians, IQRs, skewness, kurtosis, and floor and ceiling effects.

Internal consistency was evaluated using Cronbach’s α [33] and McDonald’s ω, supplemented by corrected item–rest correlations and α if item deleted. Coefficients ≥0.70 were considered acceptable and ≥0.80 good. Factorability of the 36-item psychometric core was assessed using the Kaiser–Meyer–Olkin (KMO) measure and Bartlett’s test of sphericity; KMO values of 0.60–0.69 were regarded as mediocre, 0.70–0.79 as middling, 0.80–0.89 as meritorious, and ≥0.90 as marvelous, following Kaiser. Exploratory factor analysis (EFA) used maximum-likelihood extraction with promax rotation. Factor retention was primarily guided by parallel analysis [34], supported by the scree plot and theoretical interpretability. Because the RO-ACNQ was developed around four prespecified conceptual domains, a four-factor solution was additionally examined to assess correspondence with the hypothesized structure. Items with loadings <0.40, communalities <0.20, substantial cross-loadings, or corrected item–rest correlations <0.30 were considered for refinement. EFA and the primary CFA models were estimated from Pearson product-moment correlations with maximum-likelihood estimation, a choice supported for five-category items with the observed skewness and kurtosis values (within ±2) [35]. Because the items are ordinal and several showed ceiling effects, all factor-analytic conclusions were additionally checked in sensitivity analyses based on the polychoric correlation matrix, using minimum-residual extraction for the EFA and unweighted least-squares estimation for the CFA [36].

Based on the combined EFA and item-level reliability diagnostics, a refined measurement model was derived and evaluated using confirmatory factor analysis (CFA). Model fit was assessed using the χ^2^/df ratio, comparative fit index (CFI), Tucker–Lewis index (TLI), root-mean-square error of approximation (RMSEA), and standardized root-mean-square residual (SRMR). Reference values were χ^2^/df < 3, CFI and TLI ≥ 0.95, RMSEA ≤ 0.06, and SRMR ≤ 0.08. Because model refinement and CFA evaluation were performed within the same analytical dataset, the refined CFA was considered an internal model evaluation rather than independent confirmation.

Convergent validity was examined using standardized factor loadings, composite reliability (CR), and average variance extracted (AVE), with CR ≥ 0.70 and AVE ≥ 0.50 used as reference criteria. Discriminant validity was evaluated using the Fornell–Larcker criterion and the heterotrait–monotrait (HTMT) ratio, with HTMT < 0.85 considered supportive of construct differentiation [37].

Known-groups benchmark analyses compared refined domain scores according to academic profile (Medicine/Pharmacy vs. other fields), sex (female vs. male), and antioxidant-cosmetic use (users vs. non-users/unsure) using Mann–Whitney *U* tests. Results are reported as medians with IQRs and rank-biserial correlations (*r*rb) as effect-size estimates. Simulation-based test–retest reliability was evaluated in the matched synthetic retest dataset using two-way random-effects, absolute-agreement, single-measure intraclass correlation coefficients (ICC) with 95% confidence intervals and Spearman rank correlations.

Structural equation modeling evaluated the prespecified Knowledge–Attitudes–Intention pathway, including a direct Knowledge–Intention path [29,36]. Standardized direct and indirect coefficients and overall model-fit indices were reported. Given the simulation-based and cross-sectional design, structural coefficients were interpreted as methodological benchmarks rather than causal or mediation effects.

Preliminary empirical performance of the refined model was evaluated separately in the observed pilot sample (*n* = 56) by estimating domain-level internal consistency, reported as Cronbach’s α with Feldt 95% confidence intervals in view of the small sample size. Refined domain scores were also compared between the observed pilot sample and synthetic benchmark using Mann–Whitney *U* tests with rank-biserial correlations. Item-level response distributions were compared using total variation distance (TVD), χ^2^ tests, and Cramér’s *V*, with Benjamini–Hochberg false-discovery-rate correction for multiple comparisons [38]. To reduce compositional differences, distributional comparisons were additionally performed against a synthetic subgroup matched to the observed participants by academic profile and study year (Medicine/Pharmacy, first year; *n* = 113).

Finally, exploratory associations between refined domain scores, self-rated skin health, and perceived stress were assessed using Spearman rank correlations. These analyses were considered exploratory and were not used to determine the final measurement structure.

### 2.4. Ethical Considerations

The study was conducted in accordance with the principles of the Declaration of Helsinki and good practice guidelines for biomedical research. Ethical approval was obtained from the Scientific Research Ethics Committee of the “Victor Babeș” University of Medicine and Pharmacy Timișoara (Approval No. 105/11 October 2024). Participation in the observed pilot component was voluntary, and informed consent was obtained from all participants prior to questionnaire completion. Data were collected anonymously through an online questionnaire, with no directly identifying personal information recorded. The synthetic component involved computer-generated data and therefore did not involve human participants or additional ethical risk.

## 3. Results

### 3.1. Dataset and Sample Characteristics

The analytical dataset comprised 1013 records, including 957 synthetic benchmark profiles (94.5%) and 56 observed pilot participants (5.5%). Synthetic profiles were distributed nearly equally across the four modeled university centers (UMFT, *n* = 240; UVT, UAV, and UMFCD, *n* = 239 each). Overall dataset characteristics reflected the prespecified benchmark composition: 67.5% of records were female, 77.9% represented urban residence, 82.0% bachelor-level students, and 49.7% Medicine/Pharmacy students. Median age was 21 years (IQR 20–24).

Within the observed pilot sample, all 56 participants were first-year Medicine/Pharmacy students; 71.4% were female, and the median age was 20 years (IQR 19–20). Descriptive characteristics of the complete analytical dataset are summarized in Table 2 and should be interpreted in the context of its predominantly simulation-based composition.

### 3.2. Item Distributions and Internal Consistency of the a Priori Domains

Across the analytical dataset, core-item medians ranged from 3 to 4, with IQRs generally spanning two response categories. Item distributions showed limited asymmetry (skewness, −0.79 to 0.08) and kurtosis values within ±2. Although Shapiro–Wilk tests indicated departures from normality for all core items and domain scores (all *p* < 0.001), floor effects were limited, whereas ceiling effects exceeding 30% were observed for several Knowledge, Attitudes, and Intention items (Table 3).

In the simulation-based benchmark, internal consistency was good for Knowledge (α = 0.886, ω = 0.887) and Intention (α = 0.845, ω = 0.846) and acceptable for Nutrition/Lifestyle (α = 0.778). In contrast, the a priori 10-item Attitudes domain showed low internal consistency (α = 0.475, ω = 0.522), with a mean inter-item correlation of 0.09. Corrected item–rest correlations identified weakly performing items within Attitudes, particularly Q45, Q46, Q47, Q49, and Q50 (*r*ir = 0.02–0.08). Q51 also showed a suboptimal item–rest correlation (*r*ir = 0.20). Within Nutrition/Lifestyle, Q27 (*r*ir = 0.05) and Q31 (*r*ir = 0.19) showed similarly weak associations with their hypothesized domain. These eight items (*r*ir < 0.30) were therefore flagged for further evaluation in the dimensionality analysis. The complete item-level correlation matrix is provided in Supplementary Table S1.

### 3.3. Dimensionality (Exploratory Factor Analysis)

**Figure 1:**
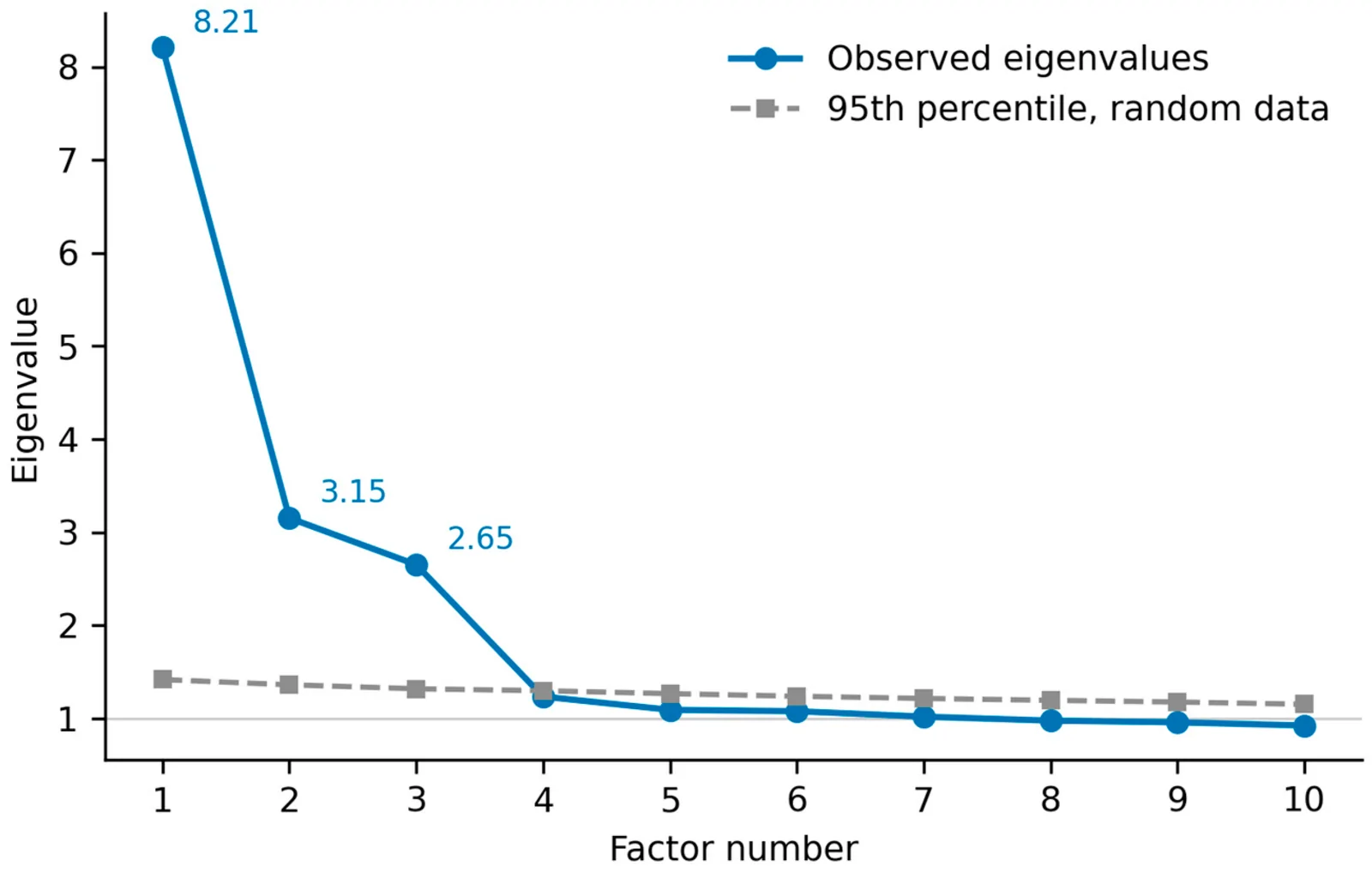
Parallel analysis scree plot (N = 1013). Observed eigenvalues (solid line) are compared with the 95th percentile of eigenvalues obtained from 100 random datasets of the same dimensions (dashed line). Three factors were retained. The analytical dataset pools 957 synthetic and 56 observed records.

The 36-item psychometric core showed excellent factorability (KMO = 0.943; Bartlett’s test of sphericity: χ^2^(630) = 10,825, *p* < 0.001). Parallel analysis supported retention of three factors: the first three observed eigenvalues (8.21, 3.15, and 2.65) exceeded the corresponding 95th-percentile random eigenvalues (1.42, 1.36, and 1.32), whereas the fourth observed eigenvalue (1.23) was below its random-data threshold (1.30) (Figure 1).

**Figure 2:**
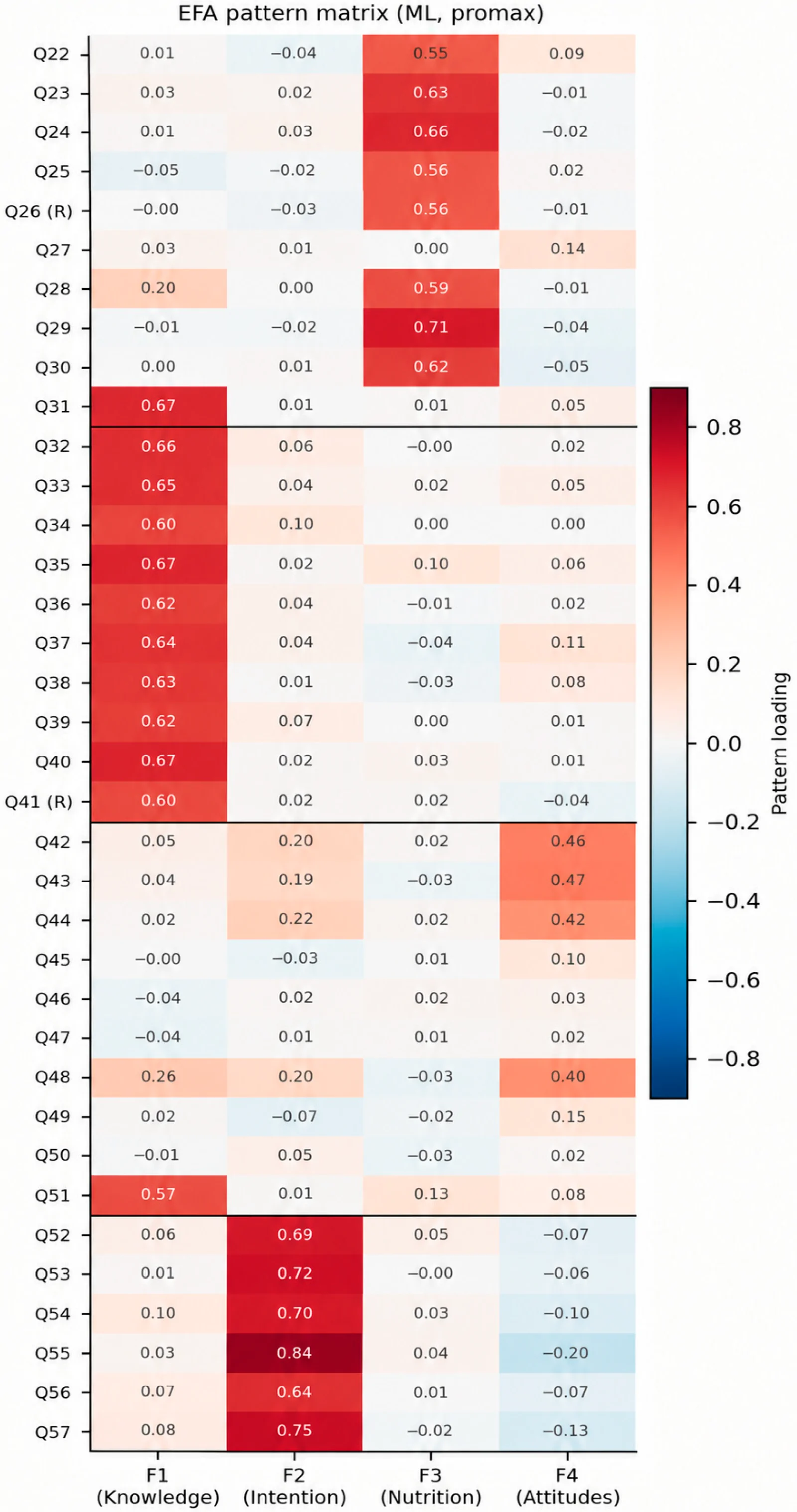
Exploratory factor analysis pattern matrix for the 36 candidate RO-ACNQ items. Maximum-likelihood extraction with promax rotation was used. Rows are grouped according to the a priori domains: Nutrition/Lifestyle, Knowledge, Attitudes, and Intention. Q45–Q47, Q49, and Q50 showed no salient loadings; Q27 was non-loading, whereas Q31 and Q51 loaded on the Knowledge factor. (R), reverse-scored item. Estimates are based on the complete analytical dataset (N = 1013: 957 synthetic and 56 observed records).

To examine correspondence with the prespecified four-domain structure, a four-factor promax solution was additionally evaluated (Figure 2). Knowledge (loadings 0.60–0.67), Intention (0.64–0.84), and Nutrition/Lifestyle (0.55–0.71) showed coherent factor-loading patterns. In contrast, the hypothesized Attitudes factor was primarily represented by Q42, Q43, Q44, and Q48 (loadings 0.40–0.47). Five items addressing skepticism, information uncertainty, price, and marketing influences (Q45–Q47, Q49, and Q50) showed negligible communalities and no salient factor loadings.

Q31 (perceived effects of stress/diet on skin) and Q51 (relative importance of lifestyle versus cosmetics) loaded on the Knowledge factor (0.67 and 0.57, respectively), rather than their hypothesized domains, whereas Q27 (supplement use) showed no salient loading. The four-factor solution explained 34.2% of the common variance, compared with 32.5% for the three-factor solution. These findings informed the subsequent evaluation of the refined measurement model. Sensitivity analyses on the polychoric correlation matrix reproduced this structure. Polychoric correlations exceeded their Pearson counterparts by 0.02 on average (maximum absolute difference 0.065), the four-factor solution explained 38.1% of the common variance, and Tucker congruence coefficients between the Pearson-based and polychoric-based factors were 0.998–1.000. The same items were flagged: Q45–Q47, Q49, and Q50 again showed negligible communalities, Q27 remained non-loading (communality 0.03), and Q31 and Q51 again loaded on the Knowledge factor (0.72 and 0.61). The four retained Attitudes items loaded 0.40–0.47, with Q48 at the 0.40 boundary. The complete polychoric pattern matrix is provided in Supplementary Table S4.

The complete EFA pattern matrix, including factor loadings and communalities, is provided in Supplementary Table S2.

### 3.4. Model Refinement and Confirmatory Factor Analysis

Based on the EFA and item-level reliability diagnostics, eight items with weak or inconsistent psychometric performance (Q27, Q31, Q45–Q47, Q49–Q51) were removed, yielding a refined 28-item, four-factor model. The Attitudes domain was thereby reduced to four items (Q42, Q43, Q44, and Q48) reflecting positive valuation and trust toward antioxidant cosmetics. Following refinement, internal consistency improved for Attitudes (α = 0.699, ω = 0.700) and Nutrition/Lifestyle (α = 0.825, ω = 0.828), while Knowledge (α = 0.886, ω = 0.887) and Intention (α = 0.845, ω = 0.846) remained unchanged. Internal consistency of the overall 28-item psychometric core was high (α = 0.891, ω = 0.889) (Table 4).

CFA showed substantially better fit for the refined model than for the a priori structure. The 36-item four-factor model showed mixed fit, with χ^2^/df = 2.53, RMSEA = 0.039, and SRMR = 0.070, but CFI (0.913) and TLI (0.907) remained below the prespecified 0.95 criterion. In comparison, the refined 28-item model showed good fit across all evaluated indices (χ^2^/df = 1.43, CFI = 0.984, TLI = 0.982, RMSEA = 0.021, SRMR = 0.033) (Table 5). The polychoric sensitivity analysis led to the same conclusion: with unweighted least-squares estimation, the refined 28-item model showed CFI = 0.990, TLI = 0.990, and RMSEA = 0.043, with standardized loadings of 0.53–0.78, whereas the a priori 36-item model again fitted poorly (CFI = 0.933, RMSEA = 0.096) (Supplementary Table S4).

In the refined model, all standardized factor loadings were statistically significant (*p* < 0.001) and ranged from 0.54 to 0.74 (Table 6). Composite reliability was acceptable for all four domains (CR = 0.70–0.89). AVE ranged from 0.37 to 0.48 and remained below the conventional 0.50 criterion, indicating that convergent validity was not fully established despite acceptable composite reliability and consistently significant factor loadings.

### 3.5. Convergent and Discriminant Validity

Composite reliability met the prespecified ≥0.70 criterion for all four refined domains: Knowledge (CR = 0.89), Intention (CR = 0.85), Nutrition/Lifestyle (CR = 0.83), and Attitudes (CR = 0.70). AVE ranged from 0.37 to 0.48 and remained below the conventional 0.50 threshold for all domains, indicating only partial support for convergent validity despite acceptable composite reliability and significant factor loadings.

Discriminant validity was supported by the HTMT criterion, with all ratios ≤0.74 and therefore below the prespecified 0.85 threshold (Table 7). The Fornell–Larcker criterion was also satisfied for most domain pairs. The exception was Attitudes–Intention, for which the inter-factor correlation (r = 0.74) exceeded the square roots of the corresponding AVEs (0.61 and 0.69), indicating substantial overlap between these two constructs. Thus, discriminant validity was generally supported, although differentiation between Attitudes and Intention warrants further evaluation in the prospective validation sample.

### 3.6. Known-Groups Validity

**Figure 3:**
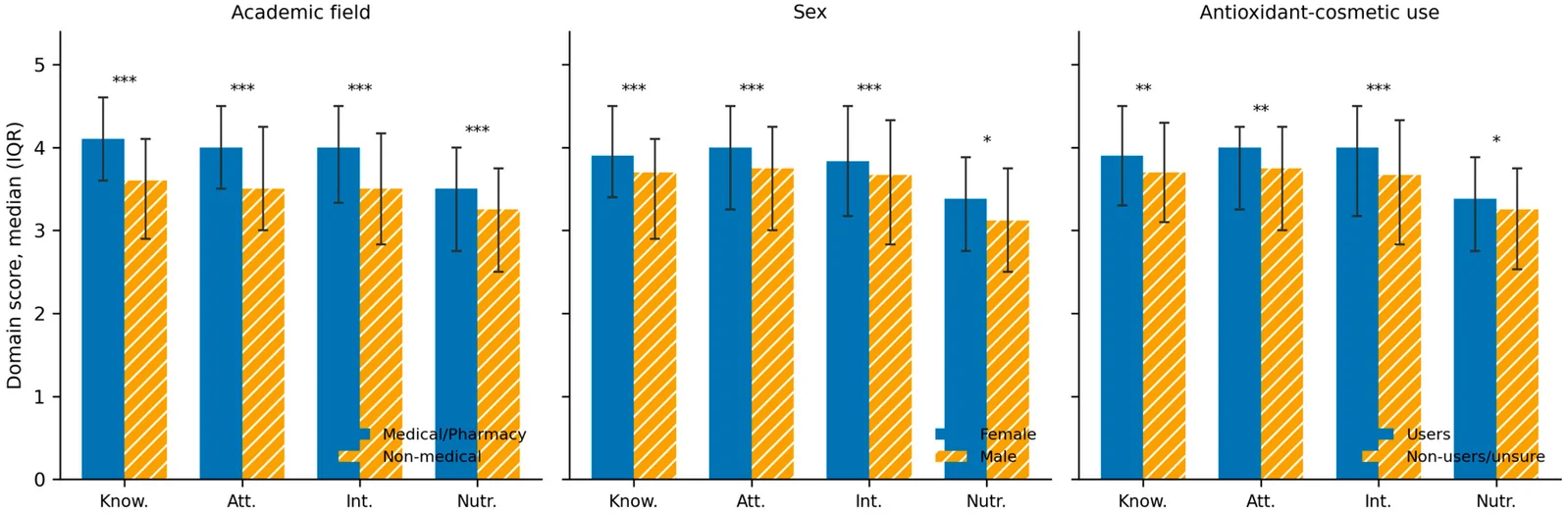
Known-groups comparisons across the refined RO-ACNQ domains. Median domain scores with interquartile ranges according to academic field, sex, and antioxidant-cosmetic use. Between-group comparisons were performed using Mann–Whitney *U* tests. * *p* < 0.05; ** *p* < 0.01; *** *p* < 0.001. Know., Knowledge; Att., Attitudes; Int., Intention; Nutr., Nutrition/Lifestyle. Comparisons are based on the complete analytical dataset (N = 1013: 957 synthetic and 56 observed records).

Known-groups comparisons within the analytical dataset showed differences in the prespecified directions (Figure 3, Table 8). Medicine/Pharmacy profiles had higher scores than non-medical profiles across all four refined domains. The largest difference was observed for Knowledge (median 4.10, IQR 3.60–4.60 vs. 3.60, IQR 2.90–4.10; *p* < 0.001; *r*rb = 0.38), followed by Attitudes (*r*rb = 0.30) and Intention (*r*rb = 0.27); the difference in Nutrition/Lifestyle was smaller (*r*rb = 0.16).

Female profiles also showed higher scores than male profiles across all domains, with effect sizes ranging from *r*rb = 0.09 for Nutrition/Lifestyle to 0.23 for Knowledge. Antioxidant-cosmetic users scored higher than non-users/unsure respondents across all domains, although effect sizes were small (*r*rb = 0.09–0.17), with the largest difference observed for Intention (*r*rb = 0.17, *p* < 0.001). Overall, these simulation-dominated benchmark comparisons reproduced the prespecified known-groups patterns, while empirical confirmation will require evaluation in the prospective multicenter sample.

### 3.7. Simulation-Based Test–Retest Reliability Benchmark

**Figure 4:**
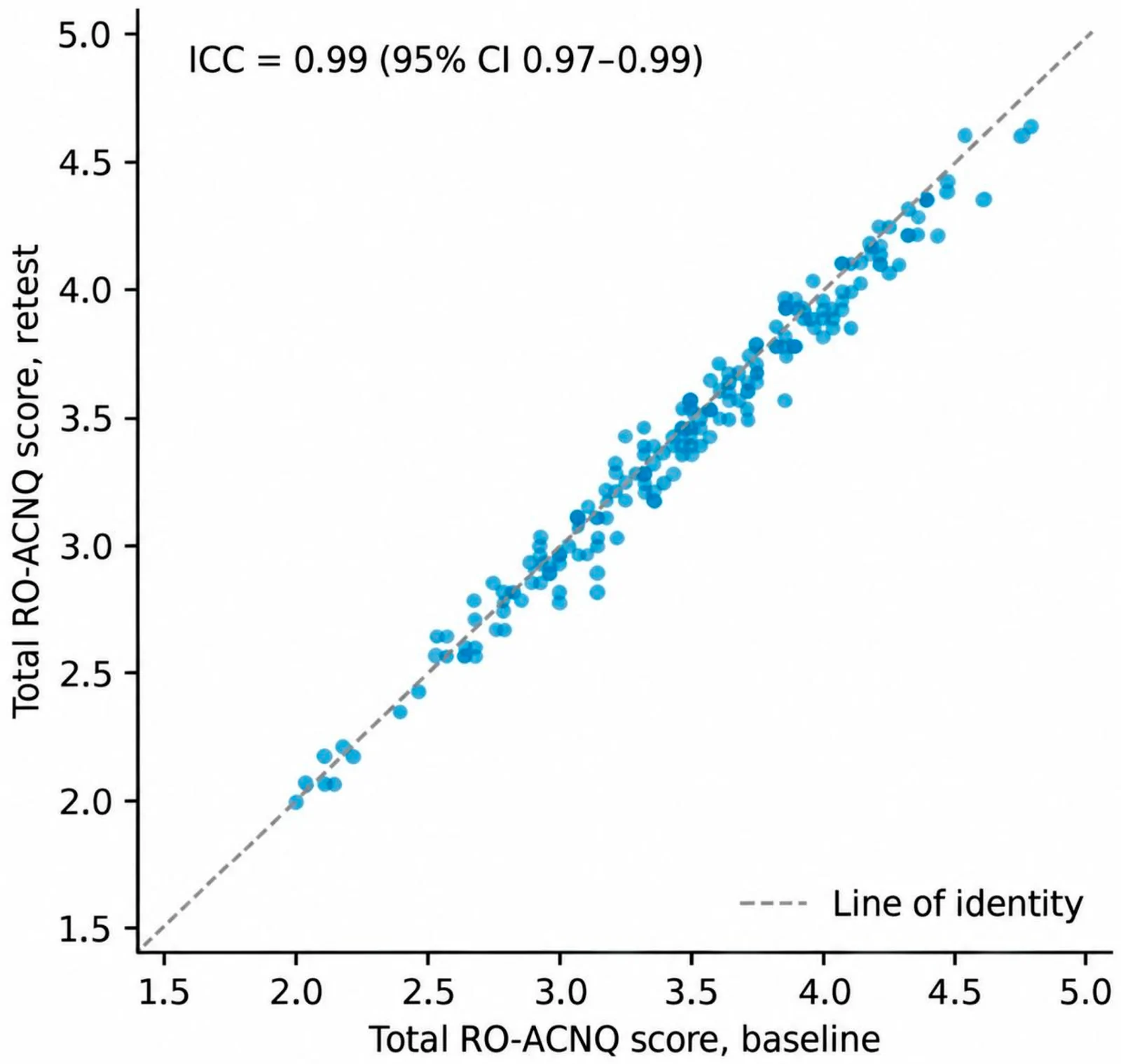
Simulation-based test–retest agreement for the overall refined RO-ACNQ score. Baseline and retest scores are shown for the matched synthetic retest dataset (*n* = 200); the dashed line represents the line of identity. The analysis provides a benchmark of the scoring and reliability pipeline rather than empirical evidence of temporal stability.

In the matched synthetic retest dataset (*n* = 200), benchmark test–retest reliability was high across all refined domains. ICCs ranged from 0.97 to 0.98, with corresponding Spearman correlations of 0.96–0.98 (Table 9). The overall 28-item score showed an ICC of 0.99 (95% CI 0.97–0.99) and a Spearman correlation of 0.99. Figure 4 illustrates the close agreement between baseline and retest total scores.

Because the retest dataset was generated with prespecified response perturbation, these estimates represent a simulation-based benchmark of the scoring and reliability-analysis procedures rather than empirical evidence of temporal stability. Test–retest reliability therefore requires confirmation in prospectively collected participant data.

### 3.8. Structural Model of the Knowledge–Attitudes–Intention Pathway

**Figure 5:**
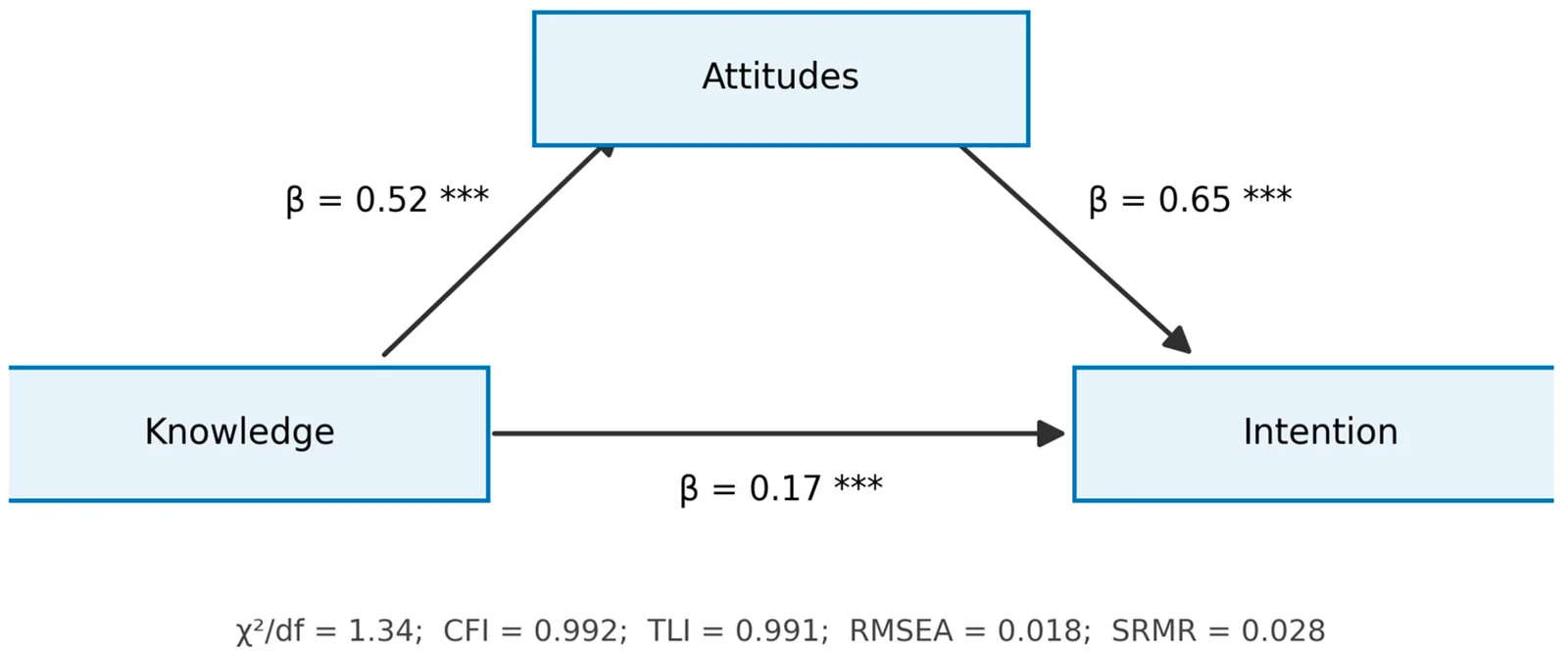
Simulation-based structural model of the Knowledge–Attitudes–Intention pathway. Standardized path coefficients are shown for the analytical dataset; measurement components are omitted for clarity. *** *p* < 0.001. The model represents a methodological benchmark of the prespecified structural relationships rather than an empirically established causal pathway. The analytical dataset comprises 957 synthetic and 56 observed records.

The prespecified Knowledge–Attitudes–Intention structural model showed good fit (χ^2^/df = 1.34, CFI = 0.992, TLI = 0.991, RMSEA = 0.018, SRMR = 0.028). Knowledge was positively associated with Attitudes (β = 0.52, *p* < 0.001), which in turn showed a strong positive association with Intention (β = 0.65, *p* < 0.001). The direct Knowledge–Intention path was weaker but remained statistically significant (β = 0.17, *p* < 0.001). The standardized indirect association between Knowledge and Intention through Attitudes was 0.34 (Figure 5).

These simulation-based findings indicate that the refined measurement and structural framework recovered the prespecified pattern of relationships among Knowledge, Attitudes, and Intention. The pathway should therefore be regarded as a structural benchmark requiring empirical evaluation in the prospective multicenter sample rather than evidence of causal or mediating effects.

### 3.9. Preliminary Empirical Evaluation in the Observed Pilot Sample

The refined 28-item structure showed encouraging internal consistency in the observed pilot sample (*n* = 56). Cronbach’s α was 0.84 (Feldt 95% CI 0.76–0.89) for Knowledge, 0.78 (95% CI 0.67–0.86) for Attitudes, 0.89 (95% CI 0.84–0.93) for Intention, and 0.84 (95% CI 0.77–0.90) for Nutrition/Lifestyle, with α = 0.91 (95% CI 0.87–0.94) for the overall psychometric core. Given the sample size, these coefficients are estimated imprecisely, and the wide confidence intervals should temper their interpretation. Notably, the refined four-item Attitudes domain showed higher internal consistency in the observed sample (α = 0.78) than in the synthetic benchmark (α = 0.70) (Table 10).

**Figure 6:**
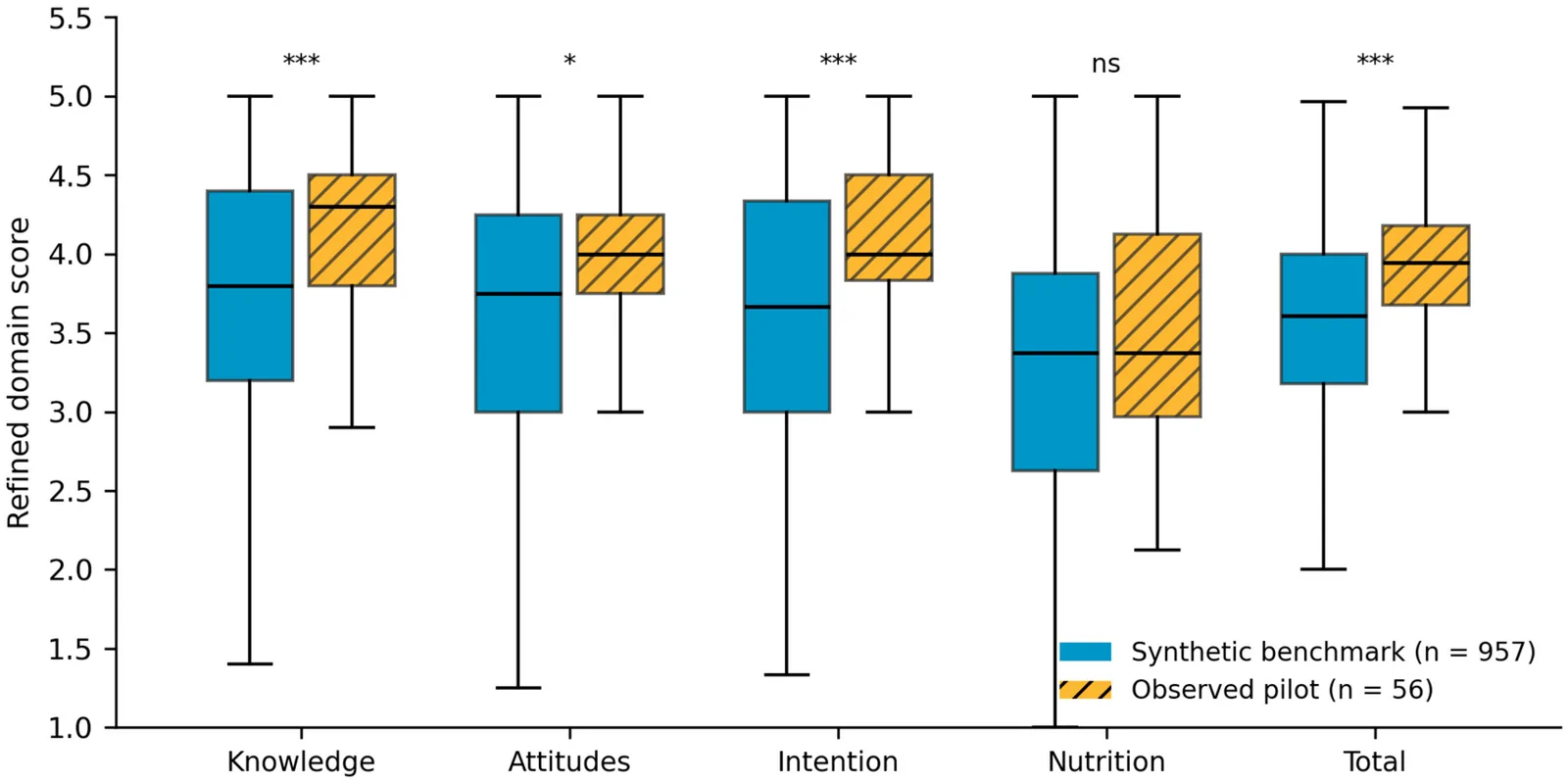
Refined RO-ACNQ domain and overall psychometric-core scores according to data source. Box plots show medians, interquartile ranges, and whiskers extending to 1.5 × IQR. Comparisons were performed using Mann–Whitney *U* tests: * *p* < 0.05; *** *p* < 0.001; ns, not significant.

Compared with the full synthetic benchmark, observed participants had higher Knowledge scores (median 4.30, IQR 3.80–4.50 vs. 3.80, IQR 3.20–4.40; *p* < 0.001, *r*rb = 0.27), Intention scores (4.00, IQR 3.83–4.50 vs. 3.67, IQR 3.00–4.33; *p* < 0.001, *r*rb = 0.27), and Attitudes scores (4.00, IQR 3.75–4.25 vs. 3.75, IQR 3.00–4.25; *p* = 0.029, *r*rb = 0.17). The overall psychometric-core score was also higher in the observed sample (3.95, IQR 3.68–4.18 vs. 3.61, IQR 3.18–4.00; *p* < 0.001, *r*rb = 0.34). Nutrition/Lifestyle scores did not differ significantly between data sources (*p* = 0.076, *r*rb = 0.14) (Table 10, Figure 6).

To account for differences in sample composition, item-level response distributions were additionally compared with a synthetic subgroup matched to the observed participants by academic profile and study year (Medicine/Pharmacy, first year; *n* = 113). Across 64 single-response questions, the median total variation distance (TVD) was 18.9 percentage points (range 0–42.6). Forty-four questions showed TVD ≥15 percentage points, and 21 showed both a maximum category difference ≥10 percentage points and a false-discovery-rate-adjusted *q* < 0.05. The largest discrepancies involved perceived influence of diet on skin health (TVD = 42.6), antioxidant supplement use (TVD = 35.2), and perceived importance of antioxidant products (TVD = 34.2).

Overall, the observed pilot findings provided preliminary empirical support for the internal consistency of the refined RO-ACNQ domains while also identifying differences between observed and simulated response distributions that warrant reassessment as the prospective multicenter sample expands.

The complete item-by-item comparison of observed and synthetic response distributions, including TVD, χ^2^ tests, Cramér’s *V*, and FDR-adjusted *q* values, is provided in Supplementary Table S3.

### 3.10. Exploratory Associations with Perceived Skin Health and Stress

Exploratory Spearman correlation analyses showed no meaningful associations between the refined RO-ACNQ domain scores and self-rated skin health or perceived stress in the analytical dataset. Correlation coefficients were negligible across all comparisons (|ρ| ≤ 0.05), and none reached statistical significance (all *p* ≥ 0.139).

Given that associations with skin health and perceived stress were not specified in the synthetic data-generating model, these pooled estimates primarily reflect the structure of the simulation-based benchmark and should not be interpreted as evidence of an absence of associations in university students. Empirical evaluation of these relationships was therefore considered exploratory and will require confirmation in the prospective multicenter sample.

## 4. Discussion

This study presents the development and initial psychometric evaluation of the RO-ACNQ, to our knowledge the first Romanian multidimensional instrument specifically designed to integrate antioxidant-related knowledge, attitudes, nutrition/lifestyle behaviors, antioxidant-cosmetic use, and behavioral intention among university students. The original 36-item psychometric core was refined to a more coherent 28-item, four-factor structure showing good internal consistency and good global model fit in an internal, same-sample CFA evaluation, although convergent validity was only partially supported. The findings also revealed substantial proximity between Attitudes and Behavioral Intention and reproduced the prespecified Knowledge–Attitudes–Intention pathway within the simulation-based benchmark. Importantly, the refined domains demonstrated encouraging internal consistency in the observed pilot sample. However, differences between observed and synthetic response distributions emphasize that simulation-based psychometric benchmarking can support instrument development and analytical stress-testing but cannot substitute for empirical validation. Similar iterative refinement has been applied in contemporary instruments for young adults, including the Young Adult Nutrition Literacy Tool, in which item generation, expert review, exploratory analysis, item reduction, CFA, and criterion validation were sequentially integrated [38].

The principal refinement concerned the Attitudes domain. Its original 10-item formulation combined positive valuation and trust in antioxidant cosmetics with conceptually distinct considerations related to safety, information uncertainty, marketing, price, and packaging. Five of these items showed negligible communalities and no salient factor loadings, contributing to low internal consistency (α = 0.475). Restricting the domain to four coherent valuation/trust items increased reliability to approximately 0.70 and, together with removal of three additional structurally inconsistent items, contributed to the substantially improved fit of the 28-item model. This differentiation is plausible because cosmetic-related behavior encompasses distinct dimensions including product knowledge, perceived risks, purchasing considerations, ingredients, and attitudes toward product use [23]. Similarly, recent nutrition-instrument research has treated knowledge and attitudes as distinct constructs within a Knowledge–Attitude–Behavior framework [39].

The excluded RO-ACNQ items should therefore not necessarily be regarded as irrelevant; instead, they may represent a distinct critical-appraisal or antioxidant-skepticism construct. This interpretation is compatible with reported heterogeneity in consumer awareness and attitudes toward organic cosmetics [40]. Research on natural personal-care products has likewise shown that attitudes are influenced by health consciousness, environmental concerns, and reasons for product use and are subsequently associated with purchase intention [41]. Together, these findings support distinguishing positive product valuation from skepticism, safety concerns, and marketing-related appraisal in future RO-ACNQ development.

The refined four-factor model showed good global fit in the internal, same-sample CFA evaluation (CFI = 0.984, TLI = 0.982, RMSEA = 0.021, SRMR = 0.033), with closely similar results in the polychoric sensitivity analysis (Supplementary Table S4), but construct validity requires more cautious interpretation. Composite reliability met the prespecified criterion for all domains and all retained items had significant standardized loadings; however, AVE values of 0.37–0.48 remained below the conventional 0.50 threshold. Convergent validity should therefore be considered only partially supported, particularly for the four-item Attitudes domain. Contemporary validation studies similarly emphasize combining multiple psychometric criteria rather than relying on internal consistency alone. The Young Adult Nutrition Literacy Tool, for example, combined EFA, CFA, item-response analyses, and criterion validity [38], while validation of the Arabic Revised General Nutrition Knowledge Questionnaire incorporated internal and test–retest reliability, construct and convergent validity, and responsiveness [42].

Discriminant validity was generally supported, with all HTMT ratios below 0.85, although the Fornell–Larcker criterion was not satisfied for Attitudes–Intention. Their relatively strong latent correlation (*r* = 0.74) is theoretically plausible because favorable product evaluation would be expected to accompany stronger behavioral intention [20]. Similar relationships among knowledge, attitudes, and purchase intention have been reported for natural cosmetics [43]. Thus, the RO-ACNQ findings support conceptual proximity between Attitudes and Intention, but their psychometric distinction should be reassessed in a larger empirical sample.

Known-groups analyses reproduced the prespecified directional patterns, with the largest difference observed for Knowledge between Medicine/Pharmacy and other academic profiles (*r*rb = 0.38). This is consistent with greater curricular exposure to nutrition, oxidative stress, and related biomedical concepts. Recent Romanian research has similarly identified gaps in antioxidant knowledge and differences according to educational background [44].

Comparable known-groups differences have been reported for nutrition-knowledge instruments, with higher scores among students with nutrition or health-related educational backgrounds [42]. The revised Australian General Nutrition Knowledge Questionnaire likewise discriminated between nutrition and engineering students [45]. Nevertheless, because the present known-groups analyses were dominated by synthetic observations, these findings represent supportive benchmark patterns rather than definitive empirical validity evidence.

The Knowledge–Attitudes–Intention model further supported the proposed conceptual structure. Knowledge predicted Attitudes (β = 0.52), which in turn showed a stronger association with Intention (β = 0.65), whereas the direct Knowledge–Intention association was smaller (β = 0.17). This pattern is consistent with behavioral models in which knowledge contributes to intention partly through evaluative processes [20] and with consumer and health-information research [43,46]. It also parallels the Knowledge–Attitude–Behavior framework, in which knowledge and attitudes are treated as distinct but behaviorally relevant constructs [39].

From an applied perspective, antioxidant knowledge alone may therefore be insufficient to influence product-use intentions unless it also shapes perceptions of usefulness, credibility, and relevance. However, because the structural model was evaluated primarily using synthetic data with prespecified construct dependencies, this pathway should be regarded as a hypothesis for prospective empirical testing rather than evidence of causal mediation.

The most important empirical support for the refined RO-ACNQ derives from the observed pilot sample. Despite comprising only 56 first-year Medicine/Pharmacy students, internal consistency was encouraging for Knowledge (α = 0.84), Attitudes (α = 0.78), Intention (α = 0.89), Nutrition/Lifestyle (α = 0.84), and the overall psychometric core (α = 0.91). Particularly relevant was the four-item Attitudes domain, which achieved α = 0.78 despite its borderline benchmark reliability. These coefficients are broadly comparable with recent multidimensional instruments developed for young adults, including the Short Nutrition Literacy Scale for Young Adults, which reported subscale α values of 0.79–0.83 [47]. A recent integrated-behavioral-model questionnaire for university students similarly reported satisfactory-to-high internal consistency across knowledge, attitudes, intention, and dietary-behavior constructs [48]. These findings provide preliminary participant-derived support for retaining the refined RO-ACNQ structure, although confirmation in the larger prospective sample remains necessary. At n = 56, however, the Feldt 95% confidence intervals around these coefficients are wide (Section 3.9), so they should be read as encouraging rather than as stable reliability estimates.

The potential relevance of the RO-ACNQ extends beyond psychometric assessment. Studies among university students have identified gaps in cosmetic-product knowledge and relationships among knowledge, product-use practices, and adverse effects [49,50]. Educational interventions have also improved cosmetic-related knowledge and skincare practices [51], while multidisciplinary education in cosmetic and dermatological sciences has improved understanding of formulation, appropriate use, adverse reactions, and dermatological concepts [52]. Following full validation, the RO-ACNQ could therefore be evaluated as an outcome measure for educational interventions addressing evidence-based antioxidant nutrition and skincare.

The observed pilot also highlighted the limitations of simulation-based benchmarking. Observed participants had higher Knowledge, Attitudes, and Intention scores than the complete synthetic benchmark, whereas Nutrition/Lifestyle did not differ significantly. Comparison with the academically harmonized synthetic subgroup yielded a median item-level TVD of 18.9 percentage points, with the largest discrepancies involving perceived diet–skin relationships, supplement use, and the perceived importance of antioxidant products. Thus, similarity in psychometric structure did not imply equivalence of response distributions. This is consistent with synthetic-data literature emphasizing that simulated datasets may not fully reproduce the dependencies and statistical properties of real-world data and should therefore be validated against observed data [53]. Concerns regarding data quality, bias, representativeness, and fidelity to real-world distributions have similarly been emphasized [54].

The very high synthetic test–retest ICCs (0.97–0.99) should be interpreted accordingly: they demonstrate recovery of the stability incorporated into the simulation rather than empirical temporal stability of the RO-ACNQ. The synthetic component is therefore best viewed as a methodological tool for analytical stress-testing and preliminary item refinement [55].

From a substantive perspective, the RO-ACNQ addresses the relatively underexplored intersection of antioxidant literacy, dietary behavior, cosmetic perceptions, and behavioral intention. University-based research indicates that general nutrition knowledge can coexist with gaps in specific areas such as nutrient sources and diet–disease relationships. This multidimensionality of nutrition knowledge has also been demonstrated more broadly [56]. Similarly, cosmetic consumption among young adults involves knowledge, perceived benefits and risks, product characteristics, professional recommendations, and purchasing practices [50], while high product use does not necessarily imply adequate safety awareness [57].

These findings support assessing antioxidant knowledge together with, but separately from, nutrition/lifestyle behavior and cosmetic-related attitudes and intentions. The relevance of incorporating an end-user dimension is also supported by recent Romanian research on natural food products. In a sensory evaluation of Romanian monofloral honeys involving young adult university students, consumer acceptability differed according to botanical origin and was associated with physicochemical characteristics, demonstrating that the objective composition of a natural product and its subjective evaluation represent complementary dimensions [14,58]. Although food acceptability and antioxidant-cosmetic behavior represent distinct contexts, this observation reinforces the broader rationale for complementing compositional and biochemical characterization of natural products with assessment of consumer perceptions and behavioral responses [14].

The RO-ACNQ is therefore well aligned with research concerning natural antioxidants for cosmetic applications. By integrating oxidative-stress literacy, antioxidant-oriented nutrition/lifestyle behaviors, attitudes toward antioxidant cosmetics, and behavioral intention, it adds an end-user perspective to the predominantly mechanistic, formulation-oriented, and efficacy-focused literature on topical and dietary antioxidants. A systematic review of 48 studies identified attitudes, consumer consciousness, values, knowledge and experience, and perceived barriers as important determinants of green-cosmetic purchase intention and behavior [59]. Similarly, attitudes toward natural cosmetics and environmental considerations have been positively associated with purchase intention, with product knowledge exerting an indirect influence through attitudes [60].

These findings support the RO-ACNQ approach of treating knowledge, attitudes, and behavioral intention as related but distinct dimensions. Following full empirical validation, the instrument may help characterize antioxidant-related knowledge and behavioral profiles and evaluate educational strategies concerning evidence-based antioxidant nutrition and skincare.

A key strength is the explicit separation between simulation-based methodological benchmarking and preliminary participant-derived evidence. The synthetic framework enabled early evaluation of the analytical pipeline and identification of problematic items, while the observed pilot provided an empirical check of the refined domains and exposed meaningful differences between simulated and real responses. This approach is consistent with recommendations that synthetic-data utility be evaluated against its intended application and, where possible, against observed data [53,54].

But the limitations are also important and should be acknowledged in order to frame interpretation of the findings. Most importantly, 957 of 1013 analytical records (94.5%) were synthetic; therefore, the EFA, CFA, known-groups analyses, structural model, and synthetic test–retest estimates cannot establish psychometric validity in human participants. EFA-based refinement and CFA were also performed within the same analytical dataset, so the good fit of the refined model represents an internal model evaluation rather than independent confirmation, and the same-sample sequence carries a risk of overfitting: the refined structure may fit less well in independent participant-derived data. The observed pilot was small (*n* = 56), restricted to first-year Medicine/Pharmacy students, and lacked university-center information, precluding independent factor analysis, measurement-invariance testing, and robust subgroup comparisons, and yielding wide confidence intervals around the observed-sample reliability coefficients. Although the primary factor analyses used Pearson correlations with maximum-likelihood estimation, polychoric-based sensitivity analyses reached the same conclusions, which mitigates, without eliminating, concerns related to the ordinal response format and the observed ceiling effects. In addition, AVE remained below 0.50 for all domains, Attitudes–Intention did not fully satisfy the Fornell–Larcker criterion, empirical test–retest reliability remains unknown, and all outcomes were self-reported without objective measures of antioxidant exposure or skin status. Accordingly, the present findings provide preliminary support for the feasibility and reliability of the refined RO-ACNQ rather than definitive validation.

The refined 28-item RO-ACNQ should next be validated prospectively in the planned four-center university sample, with its factor structure re-estimated entirely from participant-derived data and, where sample size permits, independent EFA and CFA subsets. Empirical test–retest reliability, measurement invariance across sex and academic profile, convergent and discriminant validity, and known-groups validity should also be reassessed. Particular attention should be given to expanding the Attitudes domain and evaluating whether the excluded skepticism, safety, marketing, information, price, and packaging items constitute a separate critical-appraisal construct. Future longitudinal and intervention studies should ultimately determine whether RO-ACNQ scores correspond to actual dietary and cosmetic behaviors and whether changes in antioxidant knowledge translate into changes in attitudes, intentions, and evidence-based antioxidant practices.

## 5. Conclusions

The RO-ACNQ is a promising multidimensional instrument designed to assess antioxidant-related knowledge, attitudes, nutrition/lifestyle behaviors, and behavioral intention toward antioxidant cosmetics among university students. In this simulation-based pilot augmented by an observed participant sample, psychometric refinement reduced the original 36-item core to a more coherent 28-item, four-factor structure comprising Knowledge, Attitudes, Behavioral Intention, and Nutrition/Lifestyle.

The refined model showed good internal consistency and good global fit within the benchmark dataset, although convergent validity remained only partially supported and the conceptual proximity between Attitudes and Intention warrants further evaluation. Importantly, the refined domains also indicated encouraging internal consistency in the observed pilot sample (α = 0.78–0.89; total α = 0.91), providing preliminary participant-derived support for the proposed structure. Differences between synthetic and observed response distributions further confirmed that simulation-based benchmarking is useful for instrument refinement and analytical stress-testing but cannot replace empirical psychometric validation.

Prospective multicenter validation in a larger and more diverse sample is therefore required to confirm the factor structure, validity, test–retest reliability, and measurement invariance of the RO-ACNQ. If confirmed, the instrument may provide a culturally relevant framework for investigating antioxidant literacy and its relationship with nutrition, skincare perceptions, and behavioral intentions, and for evaluating educational strategies related to evidence-based antioxidant nutrition and cosmetic use.

## Figures and Tables

**Table 1:** Structure of the Romanian Antioxidant Cosmetics and Nutrition Questionnaire (RO-ACNQ).

Section	Domain/Hypothesized Factor	Items	Response Format	Representative Content
A	Sociodemographic and lifestyle characteristics (descriptive)	10	Categorical/ordinal	Sex, age, academic field and year, residence, smoking, alcohol, sleep, physical activity, perceived stress
B	Cosmetic behaviors and antioxidant product use (descriptive)	6	Categorical/multiple choice	Antioxidant cosmetic use, ingredients, frequency, influences, reasons for use, adverse reactions
C	Dietary and supplement descriptors (descriptive)	5	Categorical/multiple choice; ordinal 1–5	Daily fruit/vegetable intake, nine-row food-frequency matrix (single item), supplement use and types, perceived diet–skin influence
C	Nutrition and antioxidant dietary behaviors (Factor 4)	10	Frequency Likert 1–5	Fruit and vegetables, antioxidant-rich foods, green tea, ultra-processed foods (R), supplements, diet and hydration
D	Knowledge of oxidative stress and antioxidants (Factor 1)	10	Agreement Likert 1–5	Free radicals, skin aging, UV exposure, antioxidant effects, smoking, SPF, sleep, pollution, self-rated knowledge (R)
E	Attitudes and perceptions toward antioxidant cosmetics (Factor 2)	10	Agreement Likert 1–5	Importance, trust, natural ingredients, adverse effects, professional advice, price, packaging, lifestyle
F	Behavioral intention and purchase (Factor 3)	6	Agreement Likert 1–5	Intention to use and recommend, interest in learning, professional recommendations, willingness to pay
G	Perceived skin health (descriptive)	4	Categorical + Likert 1–5	Self-rated skin health, skin conditions, perceived contributing factors, lifestyle support

The 36 Likert-type items in Sections C–F constitute the psychometric core evaluated in this study. (R) denotes reverse-scored items. Factor numbering refers to the four hypothesized latent dimensions evaluated by exploratory and confirmatory factor analyses. Item counts refer to numbered questions; the Q18 food-frequency matrix is counted as one item with nine rated rows (expanded to separate variables in the analytical database).

**Table 2:** Characteristics of the unified pilot sample (N = 1013).

Characteristic	*n* (%)
Data source	
Synthetic benchmark	957 (94.5)
Observed pilot (Microsoft Forms)	56 (5.5)
University center	
UMFT	240 (23.7)
UVT	239 (23.6)
UAV Arad	239 (23.6)
UMF Carol Davila	239 (23.6)
Observed pilot (center not recorded)	56 (5.5)
Sex	
Female	684 (67.5)
Male	329 (32.5)
Age (years), median (IQR)	21 (20–24)
Residence	
Urban	789 (77.9)
Rural	224 (22.1)
Study level	
Bachelor	831 (82.0)
Master	137 (13.5)
Doctoral	45 (4.4)
Academic field	
Medicine/Pharmacy	503 (49.7)
Social sciences	214 (21.1)
Exact sciences	123 (12.1)
Biological sciences	113 (11.2)
Other	60 (5.9)
Perceived stress	
Very low	36 (3.6)
Low	156 (15.4)
Moderate	457 (45.1)
High	274 (27.0)
Very high	90 (8.9)
Use of antioxidant cosmetics	
Yes	583 (57.6)
No	256 (25.3)
Unsure	174 (17.2)
Antioxidant supplement use (yes)	398 (39.3)
Daily fruit/vegetable intake (yes)	641 (63.3)
Adverse reaction to cosmetics (yes)	157 (15.5)
Self-rated skin health	
Very poor	40 (3.9)
Poor	121 (11.9)
Moderate	409 (40.4)
Good	378 (37.3)
Very good	65 (6.4)

University-center allocation applies only to the synthetic benchmark profiles; center was not recorded for the observed pilot participants. Percentages are calculated using the complete analytical dataset (N = 1013), unless otherwise indicated. IQR, interquartile range; UMFT, “Victor Babeș” University of Medicine and Pharmacy Timișoara; UVT, West University of Timișoara; UAV, “Aurel Vlaicu” University of Arad; UMFCD, “Carol Davila” University of Medicine and Pharmacy Bucharest.

**Table 3:** Item-level distributional characteristics and internal-consistency diagnostics for the a priori RO-ACNQ domains. Estimates are based on the complete analytical dataset (N = 1013: 957 synthetic and 56 observed records) and are therefore dominated by the synthetic benchmark.

Item	Mdn	IQR	Skew.	Kurt.	Floor %	Ceil. %	*r*_ir_	α (–)
Knowledge (Factor 1)								
Q32	4	3–5	−0.67	−0.42	2.6	35.3	0.64	0.873
Q33	4	3–5	−0.65	−0.45	2.0	34.1	0.63	0.874
Q34	4	3–5	−0.66	−0.40	4.1	33.4	0.58	0.877
Q35	4	3–5	−0.79	−0.23	4.1	38.5	0.66	0.871
Q36	4	3–5	−0.74	−0.39	3.7	38.6	0.58	0.877
Q37	4	3–5	−0.43	−0.82	5.9	28.4	0.62	0.875
Q38	4	3–5	−0.66	−0.39	4.5	34.2	0.58	0.876
Q39	4	3–5	−0.55	−0.71	5.8	33.6	0.60	0.875
Q40	4	3–5	−0.61	−0.54	4.5	33.6	0.63	0.873
Q41 (R)	4	3–5	−0.44	−0.82	7.1	28.1	0.56	0.879
Attitudes (Factor 2)								
Q42	4	3–5	−0.60	−0.40	2.5	32.0	0.38	0.380
Q43	4	3–5	−0.40	−0.83	4.7	28.4	0.33	0.391
Q44	4	3–4	−0.38	−0.76	5.9	25.0	0.35	0.386
Q45	3	2–4	−0.21	−0.80	7.1	16.4	0.06	0.493
Q46	3	2–4	−0.04	−0.89	12.2	13.7	0.04	0.498
Q47	3	2–4	−0.07	−0.82	9.7	12.6	0.02	0.501
Q48	4	3–5	−0.76	−0.20	3.3	36.9	0.40	0.376
Q49	3	2–4	−0.04	−0.99	12.7	15.9	0.08	0.483
Q50	3	2–4	0.02	−0.86	11.0	15.0	0.04	0.497
Q51	4	3–5	−0.50	−0.84	5.0	33.8	0.20	0.441
Intention (Factor 3)								
Q52	4	3–5	−0.56	−0.57	4.8	30.9	0.61	0.821
Q53	4	3–5	−0.53	−0.69	6.9	28.3	0.61	0.820
Q54	4	3–5	−0.63	−0.62	4.6	36.1	0.63	0.818
Q55	4	3–5	−0.71	−0.48	3.4	37.5	0.65	0.811
Q56	4	2–5	−0.35	−0.97	9.4	25.7	0.56	0.831
Q57	4	3–5	−0.59	−0.60	5.1	32.1	0.62	0.817
Nutrition/Lifestyle (Factor 4)								
Q22	3	2–4	−0.14	−0.97	10.8	18.8	0.48	0.752
Q23	3	2–4	−0.19	−0.96	13.3	17.4	0.53	0.745
Q24	3	2–4	−0.15	−0.90	10.1	17.9	0.56	0.743
Q25	3	2–4	0.08	−1.12	20.7	13.5	0.47	0.755
Q26 (R)	3	2–4	−0.17	−1.12	12.3	22.2	0.47	0.755
Q27	3	2–4	0.07	−1.08	17.2	13.5	0.05	0.808
Q28	4	3–5	−0.45	−0.68	6.0	26.3	0.57	0.742
Q29	4	3–5	−0.59	−0.46	4.6	28.3	0.57	0.742
Q30	3	2–4	−0.09	−0.93	11.5	16.9	0.52	0.748
Q31	4	3–5	−0.63	−0.52	4.9	33.9	0.19	0.788

Mdn, median; IQR, interquartile range (25th–75th percentile); *r*_ir_, Spearman corrected item–rest correlation within the a priori domain; (R), reverse-scored item. Items with *r*_ir_ < 0.30 (Q27, Q31, Q45, Q46, Q47, Q49, Q50, Q51) were flagged for revision.

**Table 4:** Internal consistency of the a priori and refined RO-ACNQ models. Values are computed on the complete analytical dataset (N = 1013: 957 synthetic and 56 observed records).

Domain	A Priori Model *k*	A Priori Model α	A Priori Model ω	Refined Model *k*	Refined Model α	Refined Model ω
Knowledge	10	0.886	0.887	10	0.886	0.887
Attitudes	10	0.475	0.522	4	0.699	0.700
Intention	6	0.845	0.846	6	0.845	0.846
Nutrition/Lifestyle	10	0.778	0.793	8	0.825	0.828
Overall psychometric core	36	0.876	0.879	28	0.891	0.889

*k*, number of items; α, Cronbach’s alpha; ω, McDonald’s omega. The refined model excluded Q27, Q31, Q45–Q47, and Q49–Q51.

**Table 5:** Confirmatory factor analysis fit indices for the a priori and refined four-factor RO-ACNQ models. Models were estimated on the complete analytical dataset (N = 1013: 957 synthetic and 56 observed records).

Model	χ^2^	df	χ^2^/df	CFI	TLI	RMSEA	SRMR
A priori (36 items)	1489.0	588	2.53	0.913	0.907	0.039	0.070
Refined (28 items)	493.3	344	1.43	0.984	0.982	0.021	0.033

CFI, comparative fit index; TLI, Tucker–Lewis index; RMSEA, root-mean-square error of approximation; SRMR, standardized root-mean-square residual. Prespecified reference values were χ^2^/df < 3, CFI/TLI ≥ 0.95, RMSEA ≤ 0.06, and SRMR ≤ 0.08.

**Table 6:** Standardized confirmatory factor loadings, composite reliability (CR) and average variance extracted (AVE) for the refined 28-item model. Estimates are based on the complete analytical dataset (N = 1013: 957 synthetic and 56 observed records).

Item	Std. Loading
Knowledge (Factor 1) (CR = 0.89, AVE = 0.44)	
Q32	0.69
Q33	0.69
Q34	0.64
Q35	0.72
Q36	0.63
Q37	0.67
Q38	0.65
Q39	0.66
Q40	0.69
Q41 (R)	0.60
Attitudes (Factor 2) (CR = 0.70, AVE = 0.37)	
Q42	0.60
Q43	0.58
Q44	0.57
Q48	0.67
Intention (Factor 3) (CR = 0.85, AVE = 0.48)	
Q52	0.69
Q53	0.69
Q54	0.70
Q55	0.74
Q56	0.63
Q57	0.71
Nutrition/Lifestyle (Factor 4) (CR = 0.83, AVE = 0.38)	
Q22	0.56
Q23	0.64
Q24	0.67
Q25	0.54
Q26 (R)	0.54
Q28	0.65
Q29	0.68
Q30	0.61

(R), reverse-scored item.

**Table 7:** Discriminant validity of the refined four-factor RO-ACNQ model. Estimates are based on the complete analytical dataset (N = 1013: 957 synthetic and 56 observed records).

Factor	1	2	3	4
1. Knowledge	**0.66**	[0.49]	[0.50]	[0.31]
2. Attitudes	0.52	**0.61**	[0.74]	[0.27]
3. Intention	0.50	0.74	**0.69**	[0.22]
4. Nutrition	0.32	0.27	0.22	**0.61**

Values below the diagonal are inter-factor correlations from the refined CFA; bold diagonal values represent the square root of AVE; values above the diagonal in brackets are HTMT ratios. The Fornell–Larcker criterion is supported when the square root of AVE exceeds the corresponding inter-factor correlations; HTMT values < 0.85 were considered supportive of discriminant validity.

**Table 8:** Known-groups comparisons for the refined RO-ACNQ domain scores. Comparisons use the complete analytical dataset (N = 1013: 957 synthetic and 56 observed records) and are therefore simulation-dominated benchmark contrasts.

Domain	Group 1, Mdn (IQR)	Group 2, Mdn (IQR)	*p*	*r*_rb_
Field (Medical, *n* = 503 vs. Non-medical, *n* = 510)				
Knowledge	4.10 (3.60–4.60)	3.60 (2.90–4.10)	<0.001	0.38
Attitudes	4.00 (3.50–4.50)	3.50 (3.00–4.25)	<0.001	0.30
Intention	4.00 (3.33–4.50)	3.50 (2.83–4.17)	<0.001	0.27
Nutrition/Lifestyle	3.50 (2.75–4.00)	3.25 (2.50–3.75)	<0.001	0.16
Sex (Female, *n* = 684 vs. Male, *n* = 329)				
Knowledge	3.90 (3.40–4.50)	3.70 (2.90–4.10)	<0.001	0.23
Attitudes	4.00 (3.25–4.50)	3.75 (3.00–4.25)	<0.001	0.18
Intention	3.83 (3.17–4.50)	3.67 (2.83–4.33)	<0.001	0.14
Nutrition/Lifestyle	3.38 (2.75–3.88)	3.12 (2.50–3.75)	0.016	0.09
Cosmetic use (Users, *n* = 583 vs. Non-users/unsure, *n* = 430)				
Knowledge	3.90 (3.30–4.50)	3.70 (3.10–4.30)	0.002	0.11
Attitudes	4.00 (3.25–4.25)	3.75 (3.00–4.25)	0.007	0.10
Intention	4.00 (3.17–4.50)	3.67 (2.83–4.33)	<0.001	0.17
Nutrition/Lifestyle	3.38 (2.75–3.88)	3.25 (2.53–3.75)	0.015	0.09

Mdn, median; IQR, interquartile range; rrb*, rank-biserial correlation. Group 1 and Group 2 correspond to the order specified in each comparison; positive rrb values indicate higher scores in Group 1. Two-tailed Mann–Whitney U tests were used *.

**Table 9:** Simulation-based test–retest reliability of the refined RO-ACNQ domains and overall score (*n* = 200). Estimates are based exclusively on the matched synthetic retest dataset (n = 200); no observed participants were retested.

Domain	ICC	95% CI	Spearman *p*
Knowledge	0.98	0.98–0.99	0.98
Attitudes	0.97	0.95–0.97	0.96
Intention	0.97	0.96–0.98	0.97
Nutrition/Lifestyle	0.98	0.97–0.98	0.98
Total scale	0.99	0.97–0.99	0.99

ICC, intraclass correlation coefficient (two-way random-effects model, absolute agreement, single measures); CI, confidence interval; ρ, Spearman rank correlation. Estimates were derived exclusively from the matched synthetic retest dataset and should be interpreted as methodological benchmarks.

**Table 10:** Refined RO-ACNQ domain scores and internal consistency according to data source.

Domain	Synthetic, Mdn (IQR)	Observed, Mdn (IQR)	*p*	*r*_rb_	α Synth.	α Obs.
Knowledge	3.80 (3.20–4.40)	4.30 (3.80–4.50)	<0.001	0.27	0.89	0.84
Attitudes	3.75 (3.00–4.25)	4.00 (3.75–4.25)	0.029	0.17	0.70	0.78
Intention	3.67 (3.00–4.33)	4.00 (3.83–4.50)	<0.001	0.27	0.84	0.89
Nutrition/Lifestyle	3.38 (2.62–3.88)	3.38 (2.97–4.12)	0.076	0.14	0.83	0.84
Total scale	3.61 (3.18–4.00)	3.95 (3.68–4.18)	<0.001	0.34	0.89	0.91

Synthetic benchmark, *n* = 957; observed pilot, *n* = 56. *r*_rb_ is reported as magnitude; observed respondents scored higher on all significant contrasts. α Synth./α Obs., Cronbach’s α within the synthetic and observed subsamples (α for the total scale in the synthetic subsample = 0.89 on the pooled data).

## Data Availability

The data presented in this study are available on request from the corresponding author. The data is not publicly available due to privacy and ethical restrictions.

## Supplementary Materials

The following supporting information can be downloaded at https://www.mdpi.com/article/10.3390/antiox15101282/s1, File S1: Full RO-ACNQ instrument (Romanian original and English translation), administration instructions, and scoring syntax; Table S1: Complete item-level correlation matrix for the 36 candidate psychometric items of the RO-ACNQ (N = 1013); Table S2: Complete exploratory factor analysis pattern matrix of the RO-ACNQ (maximum-likelihood extraction, promax rotation, four-factor solution; N = 1013); Table S3: Item-by-item comparison of observed and synthetic response distributions in the harmonized Medicine/Pharmacy first-year subgroup; Table S4: Polychoric-based sensitivity analyses (exploratory factor analysis pattern matrix and confirmatory factor analysis fit).

## Abbreviations

The following abbreviations are used in this manuscript:

RO-ACNQ	Romanian Antioxidant Cosmetics and Nutrition Questionnaire
ROS	Reactive oxygen species
EFA	Exploratory factor analysis
CFA	Confirmatory factor analysis
KMO	Kaiser–Meyer–Olkin measure of sampling adequacy
CFI	Comparative fit index
TLI	Tucker–Lewis index
RMSEA	Root-mean-square error of approximation
SRMR	Standardized root-mean-square residual
CR	Composite reliability
AVE	Average variance extracted
HTMT	Heterotrait–monotrait ratio of correlations
ICC	Intraclass correlation coefficient
IQR	Interquartile range
SEM	Structural equation modeling
TVD	Total variation distance
FDR	False discovery rate
SPF	Sun protection factor
UMFT	“Victor Babeș” University of Medicine and Pharmacy Timișoara
UVT	West University of Timișoara
UAV	“Aurel Vlaicu” University of Arad
UMFCD	“Carol Davila” University of Medicine and Pharmacy Bucharest
