# Supplementary material for: Development and Pilot Validation of the Romanian Antioxidant Cosmetics and Nutrition Questionnaire (RO-ACNQ): A Simulation-Based Multicenter Benchmark with an Observed Pilot Sample
Source: Antioxidants (Basel). 2026 Oct 3;15(10):1282. doi: 10.3390/antiox15101282 (PMC13700888; doi:10.3390/antiox15101282)
Supplement: Supplementary file 1 [file antioxidants-15-01282-s001.zip › antioxidants-4559119-supplementary Tables.pdf]

Supplementary Table S1. Complete item-level correlation matrix for the 36 candidate psychometric items of the RO-ACNQ (*N* = 1013). Pearson correlations are reported above the diagonal and Spearman rank correlations below the diagonal. Q26 and Q41 are reverse-scored (R). Correlation coefficients ≥ |0.30| are shown in bold. Item order follows the a priori domains: Nutrition/Lifestyle (Q22–Q31), Knowledge (Q32–Q41), Attitudes (Q42–Q51), and Behavioral Intention (Q52–Q57).

Item	Q22	Q23	Q24	Q25	Q26	Q27	Q28	Q29	Q30	Q31	Q32	Q33	Q34	Q35	Q36	Q37	Q38	Q39	Q40	Q41	Q42	Q43	Q44	Q45	Q46	Q47	Q48	Q49	Q50	Q51	Q52	Q53	Q54	Q55	Q56	Q57
**Q22**	—	**.36**	**.36**	.30	**.32**	.06	**.37**	**.38**	**.34**	.10	.09	.10	.10	.19	.08	.10	.09	.13	.14	.09	.15	.08	.13	.01	.01	.03	.13	−.00	−.02	.20	.12	.09	.09	.08	.07	.08
**Q23**	**.35**	—	**.44**	**.34**	**.37**	.01	**.36**	**.45**	**.38**	.15	.15	.16	.12	.21	.14	.10	.12	.15	.18	.08	.13	.09	.10	.01	.04	−.02	.14	.06	−.01	.18	.15	.08	.15	.15	.08	.07
**Q24**	**.35**	**.43**	—	**.37**	**.36**	−.01	**.45**	**.45**	**.39**	.14	.12	.16	.11	.20	.14	.11	.12	.14	.13	.09	.10	.10	.11	.05	.04	−.00	.14	−.01	−.00	.21	.12	.11	.11	.13	.12	.12
**Q25**	.29	**.34**	**.37**	—	**.33**	.03	**.35**	**.34**	**.35**	.06	.06	.06	.07	.15	.07	.07	.03	.04	.08	.06	.09	.07	.10	−.02	−.06	−.02	.06	−.01	−.03	.10	.09	.07	.05	.05	.08	.04
**Q26 (R)**	**.31**	**.36**	**.35**	**.32**	—	.01	**.33**	**.38**	**.31**	.08	.11	.11	.06	.15	.07	.10	.06	.03	.10	.12	.09	.03	.05	.01	.01	−.02	.10	−.03	−.04	.17	.07	.04	.08	.07	.06	.03
**Q27**	.06	.01	−.00	.04	.01	—	.06	.05	.02	.07	.10	.03	.07	.02	.05	.10	.01	.07	.02	.05	.11	.10	.09	.00	.03	−.02	.09	.03	.05	.06	.08	.07	.02	.09	.05	.05
**Q28**	**.35**	**.35**	**.44**	**.35**	**.33**	.07	—	**.44**	**.40**	.25	.23	.24	.25	.27	.20	.20	.23	.23	.27	.23	.17	.13	.09	.00	.01	−.03	.15	.01	−.01	**.31**	.17	.12	.16	.14	.10	.15
**Q29**	**.38**	**.42**	**.44**	**.34**	**.38**	.04	**.42**	—	**.45**	.10	.09	.13	.11	.20	.09	.09	.08	.12	.11	.12	.10	.09	.11	.04	−.01	.03	.04	−.00	−.01	.20	.09	.07	.10	.11	.08	.03
**Q30**	**.34**	**.37**	**.39**	**.35**	**.30**	.02	**.40**	**.44**	—	.12	.12	.13	.11	.12	.08	.09	.11	.10	.12	.10	.08	.04	.08	−.00	.03	.02	.11	.02	.01	.18	.10	.07	.10	.09	.08	.07
**Q31**	.11	.14	.14	.07	.07	.07	.25	.10	.12	—	**.43**	**.49**	**.45**	**.51**	**.45**	**.45**	**.46**	**.43**	**.46**	**.40**	.19	.14	.16	.04	.00	−.04	.27	.01	−.00	**.45**	.24	.21	.25	.20	.18	.24
**Q32**	.09	.16	.12	.06	.11	.11	.23	.09	.12	**.43**	—	**.44**	**.44**	**.47**	**.44**	**.48**	**.46**	**.47**	**.48**	**.44**	.18	.15	.17	.01	−.03	.01	.29	.04	.00	**.44**	.25	.25	.24	.25	.23	.22
**Q33**	.12	.15	.16	.07	.11	.03	.24	.12	.14	**.47**	**.43**	—	**.45**	**.52**	**.44**	**.47**	**.43**	**.45**	**.49**	**.41**	.20	.20	.16	.01	.01	−.01	**.32**	.03	.01	**.42**	.23	.24	.26	.26	.21	.27
**Q34**	.09	.11	.10	.07	.06	.08	.25	.11	.11	**.44**	**.44**	**.44**	—	**.48**	**.39**	**.40**	**.42**	**.41**	**.44**	**.38**	.19	.15	.16	.03	−.03	−.01	.27	.04	−.01	**.38**	.27	.24	.25	.24	.25	.23
**Q35**	.19	.22	.20	.15	.15	.03	.27	.21	.13	**.49**	**.45**	**.51**	**.46**	—	**.44**	**.45**	**.48**	**.47**	**.50**	**.45**	.19	.20	.20	.03	−.02	.01	**.32**	.01	−.00	**.47**	.26	.19	.28	.24	.26	.26
**Q36**	.09	.15	.15	.07	.07	.03	.21	.09	.08	**.45**	**.43**	**.43**	**.39**	**.43**	—	**.41**	**.44**	**.43**	**.43**	**.36**	.15	.14	.14	.02	−.00	−.01	**.30**	.01	.00	**.41**	.22	.17	.27	.20	.22	.22
**Q37**	.11	.10	.11	.07	.09	.10	.21	.09	.09	**.44**	**.48**	**.46**	**.40**	**.45**	**.41**	—	**.45**	**.44**	**.47**	**.42**	.21	.22	.17	.02	−.05	−.01	**.34**	.03	.02	**.43**	.25	.23	.26	.27	.21	.25
**Q38**	.10	.13	.13	.04	.06	.02	.23	.09	.11	**.45**	**.45**	**.41**	**.40**	**.45**	**.43**	**.44**	—	**.40**	**.41**	**.37**	.19	.15	.16	.02	−.01	−.03	**.32**	.02	.01	**.42**	.23	.18	.26	.20	.20	.26
**Q39**	.13	.15	.14	.04	.03	.07	.23	.12	.11	**.44**	**.45**	**.44**	**.40**	**.46**	**.43**	**.44**	**.38**	—	**.45**	**.40**	.19	.16	.15	−.01	−.05	−.03	.30	.03	.01	**.41**	.22	.20	.29	.25	.23	.24
**Q40**	.15	.18	.13	.07	.10	.01	.27	.12	.13	**.45**	**.49**	**.48**	**.42**	**.51**	**.42**	**.47**	**.40**	**.45**	—	**.41**	.16	.16	.15	.01	.01	−.00	.28	.01	.01	**.44**	.21	.18	.25	.24	.21	.23
**Q41 (R)**	.09	.07	.09	.07	.12	.04	.24	.12	.11	**.39**	**.43**	**.40**	**.38**	**.43**	**.35**	**.41**	**.35**	**.40**	**.40**	—	.11	.11	.10	−.04	−.00	−.03	.25	−.01	−.02	**.37**	.21	.18	.17	.19	.15	.19
**Q42**	.15	.12	.09	.10	.08	.11	.16	.09	.07	.19	.18	.18	.18	.19	.13	.21	.18	.19	.16	.10	—	**.35**	**.37**	.03	.05	.03	**.39**	.05	.04	.21	**.32**	**.33**	**.31**	.30	.29	**.30**
**Q43**	.07	.08	.09	.07	.03	.10	.13	.07	.03	.14	.15	.19	.14	.20	.15	.21	.15	.15	.15	.09	**.34**	—	**.36**	.05	−.00	−.00	**.39**	.04	.00	.17	**.31**	**.32**	**.30**	.28	.28	.29
**Q44**	.13	.10	.11	.11	.05	.09	.10	.10	.08	.16	.18	.15	.15	.20	.14	.17	.17	.15	.15	.10	**.36**	**.35**	—	.06	.03	.01	**.34**	.04	.03	.17	**.32**	**.31**	**.31**	.30	.27	.28
**Q45**	.01	.00	.05	−.02	.01	−.00	−.00	.04	−.01	.05	.01	.01	.02	.04	.01	.01	.03	−.01	−.00	−.04	.04	.05	.06	—	.00	.05	.03	.03	−.03	−.01	.02	−.00	.01	.02	.04	−.01
**Q46**	.01	.03	.03	−.06	.01	.04	.01	−.01	.03	.00	−.03	.01	−.03	−.01	−.00	−.05	−.01	−.05	.01	−.01	.05	−.01	.03	−.01	—	.02	.01	.03	.01	.02	.03	.01	.01	.03	.01	.01
**Q47**	.04	−.02	−.00	−.01	−.01	−.01	−.03	.03	.02	−.03	−.00	−.00	−.02	.00	−.02	−.02	−.02	−.03	−.02	−.03	.03	−.01	.01	.05	.02	—	−.02	.04	.01	−.03	.02	.01	−.03	.02	−.01	.00
**Q48**	.13	.14	.14	.06	.09	.07	.15	.05	.11	.27	.28	**.31**	.26	**.31**	.29	**.32**	**.31**	.29	.28	.24	**.36**	**.39**	**.33**	.03	.02	−.01	—	.08	.01	**.32**	**.34**	**.32**	**.36**	**.35**	**.33**	**.36**
**Q49**	.01	.06	−.01	−.00	−.02	.04	.01	.00	.03	.02	.06	.04	.03	.01	.02	.04	.03	.04	.02	−.01	.05	.04	.04	.02	.03	.04	.07	—	.07	−.02	−.01	.02	−.02	.02	−.02	.01
**Q50**	−.02	−.01	−.01	−.03	−.04	.05	−.00	−.01	.01	.01	.00	.02	−.01	.00	.01	.01	.01	.02	.02	−.03	.03	−.00	.03	−.03	.02	.01	.02	.07	—	.02	.05	.01	.02	.03	.05	.06
**Q51**	.20	.18	.22	.10	.16	.07	**.31**	.18	.17	**.45**	**.44**	**.40**	**.37**	**.46**	**.39**	**.43**	**.40**	**.41**	**.43**	**.35**	.21	.17	.16	.00	.03	−.03	**.31**	−.01	.02	—	.26	.20	.26	.20	.25	.23
**Q52**	.11	.13	.12	.09	.06	.08	.17	.06	.09	.22	.24	.22	.27	.25	.21	.24	.21	.22	.20	.19	**.30**	.30	**.33**	.02	.04	.01	**.32**	−.00	.05	.24	—	**.49**	**.46**	**.51**	**.43**	**.47**
**Q53**	.09	.07	.12	.08	.05	.08	.13	.07	.07	.21	.25	.24	.24	.19	.18	.24	.19	.21	.18	.19	**.32**	**.32**	**.30**	.00	−.00	.01	.29	.02	.01	.20	**.48**	—	**.48**	**.50**	**.41**	**.50**
**Q54**	.08	.15	.10	.05	.07	.03	.15	.09	.10	.23	.22	.24	.24	.26	.26	.25	.24	.28	.23	.16	.29	.30	.30	.01	.01	−.03	**.34**	−.02	.01	.24	**.46**	**.48**	—	**.52**	**.47**	**.47**
**Q55**	.07	.14	.12	.05	.07	.09	.13	.10	.09	.20	.22	.25	.24	.24	.20	.27	.19	.26	.23	.18	.28	.26	.30	.02	.03	.03	**.33**	.02	.04	.20	**.50**	**.47**	**.52**	—	**.46**	**.55**
**Q56**	.07	.08	.12	.08	.05	.05	.10	.07	.08	.18	.22	.20	.25	.26	.21	.21	.19	.23	.21	.15	.28	.27	.26	.05	.01	−.01	**.32**	−.02	.04	.25	**.42**	**.41**	**.46**	**.45**	—	**.44**
**Q57**	.08	.06	.12	.04	.02	.05	.14	.02	.06	.22	.22	.27	.23	.27	.23	.24	.26	.24	.23	.20	.28	.29	.28	.02	.01	.02	**.35**	.02	.05	.23	**.47**	**.49**	**.47**	**.52**	**.43**	—

Supplementary Table S2. Complete exploratory factor analysis pattern matrix of the RO-ACNQ (maximum-likelihood extraction, promax rotation, four-factor solution; *N* = 1013). Pattern loadings and communalities (h^2) are reported, with loadings ≥ 0.40 shown in bold. Factor order: F1 (Knowledge), F2 (Behavioral Intention), F3 (Nutrition/Lifestyle), and F4 (Attitudes). KMO = 0.943; Bartlett's $\chi^2(630) = 10,825$, $p < 0.001$; total common variance explained = 34.2%. (R), reverse-scored item.

Item	Domain (a priori)	F1	F2	F3	F4	h^2
Q22	Nutrition/Lifestyle	0.01	−0.04	**0.55**	0.09	0.31
Q23	Nutrition/Lifestyle	0.03	0.02	**0.63**	−0.01	0.39
Q24	Nutrition/Lifestyle	0.01	0.03	**0.66**	−0.02	0.44
Q25	Nutrition/Lifestyle	−0.05	−0.02	**0.56**	0.02	0.31
Q26 (R)	Nutrition/Lifestyle	−0.00	−0.03	**0.56**	−0.01	0.31
Q27	Nutrition/Lifestyle	0.03	0.01	0.00	0.14	0.02
Q28	Nutrition/Lifestyle	0.20	0.00	**0.59**	−0.01	0.38
Q29	Nutrition/Lifestyle	−0.01	−0.02	**0.71**	−0.04	0.50
Q30	Nutrition/Lifestyle	0.00	0.01	**0.62**	−0.05	0.39
Q31	Nutrition/Lifestyle	**0.67**	0.00	0.01	0.05	0.45
Q32	Knowledge	**0.66**	0.06	−0.00	0.02	0.43
Q33	Knowledge	**0.65**	0.04	0.02	0.05	0.43
Q34	Knowledge	**0.60**	0.10	0.00	0.00	0.36
Q35	Knowledge	**0.67**	0.02	0.10	0.06	0.46
Q36	Knowledge	**0.62**	0.04	−0.01	0.02	0.39
Q37	Knowledge	**0.64**	0.04	−0.04	0.11	0.42
Q38	Knowledge	**0.63**	0.01	−0.03	0.08	0.41
Q39	Knowledge	**0.62**	0.07	0.00	0.01	0.39
Q40	Knowledge	**0.67**	0.02	0.03	0.01	0.45
Q41 (R)	Knowledge	**0.60**	0.02	0.02	−0.04	0.37
Q42	Attitudes	0.05	0.20	0.02	**0.46**	0.26
Q43	Attitudes	0.04	0.19	−0.03	**0.47**	0.26
Q44	Attitudes	0.02	0.22	0.02	**0.42**	0.22
Q45	Attitudes	−0.00	−0.03	0.01	0.10	0.01
Q46	Attitudes	−0.04	0.02	0.02	0.03	0.00
Q47	Attitudes	−0.04	0.01	0.01	0.02	0.00
Q48	Attitudes	0.26	0.20	−0.03	**0.40**	0.27
Q49	Attitudes	0.02	−0.07	−0.02	0.15	0.03
Q50	Attitudes	−0.01	0.05	−0.03	0.02	0.00

Q51	Attitudes	**0.57**	0.01	0.13	0.08	0.35
Q52	Intention	0.06	**0.69**	0.05	−0.07	0.48
Q53	Intention	0.01	**0.72**	−0.00	−0.06	0.52
Q54	Intention	0.10	**0.70**	0.03	−0.10	0.51
Q55	Intention	0.03	**0.84**	0.04	−0.20	0.74
Q56	Intention	0.07	**0.64**	0.01	−0.07	0.42
Q57	Intention	0.08	**0.75**	−0.02	−0.13	0.58

Supplementary Table S3. Item-by-item comparison of observed and synthetic response distributions in the harmonized Medicine/Pharmacy first-year subgroup. Observed responses were collected via Microsoft Forms (n = 56) and compared with the harmonized synthetic subgroup matched to the observed respondent profile (Medicine/Pharmacy, first year; n = 113). TVD, total variation distance, expressed in percentage points (0 = identical distributions); Max diff, largest single-category difference in percentage points; q, Benjamini–Hochberg false-discovery-rate-adjusted p value for the χ^2 test; V, Cramér's V. Rows are sorted by descending TVD. Field of study and year of study are structurally identical by design of the observed sample and are included for completeness.

**Variable**	**Item (English label)**	**TVD (pp)**	**Max Diff (pp)**	**q (FDR)**	**V**
Q21	Diet influences skin health (score 1–5)	42.6	39.6	<0.001	0.41
Q19	Antioxidant supplement use	35.1	35.1	<0.001	0.33
Q42	Antioxidant products important for skin care	34.2	32.4	0.007	0.34
Q40	Pollution contributes to oxidative skin damage	32.7	24.8	0.007	0.35
Q22	Fresh fruit consumption	30.9	20.3	0.007	0.33
Q30	Balanced diet frequency	30.7	17.2	0.007	0.33
Q44	Trust in efficacy of antioxidant cosmetics	29.0	20.2	0.024	0.29
Q53	Would recommend antioxidant products to others	29.0	23.4	0.013	0.31
Q49	Price influences product choice	28.9	14.5	0.013	0.31
Q41	Not sufficiently informed about antioxidants (R)	28.9	21.1	0.024	0.29
Q26	Ultra-processed food consumption (R)	28.9	23.3	0.007	0.33
Q18	Food frequency: green tea	28.7	28.7	0.007	0.32
Q23	Fresh vegetable consumption	26.3	18.1	0.019	0.30
Q18	Food frequency: energy drinks	26.3	22.5	0.024	0.29
Q46	Perceived over-promotion on social media	26.2	16.2	0.013	0.31
Q39	Insufficient sleep affects skin via oxidative mechanisms	25.6	15.5	0.048	0.27
Q13	Frequency of antioxidant-product use	25.5	15.5	0.044	0.25

Q61	Lifestyle supports skin health (score 1–5)	25.5	13.6	0.051	0.26
Q47	Confusing information about products	25.4	23.3	0.007	0.35
Q18	Food frequency: berries	24.4	18.0	0.051	0.27
Q43	Preference for natural antioxidant ingredients	23.7	15.8	0.097	0.24
Q32	Free radicals contribute to skin aging	22.7	17.2	0.075	0.25
Q31	Observing stress/unhealthy diet effects on skin	21.8	19.1	0.178	0.21
Q11	Use of antioxidant cosmetics	21.2	21.2	0.007	0.29
Q15	Main reason for use	20.8	12.7	0.250	0.23
Q55	Would use products recommended by dermatologist/pharmacist	20.3	16.3	0.059	0.26
Q35	Fruits and vegetables support skin health	20.1	20.1	0.072	0.25
Q52	Intention to use antioxidant products in the future	19.9	19.9	0.053	0.26
Q57	Would use Romanian/natural products with scientific evidence	19.9	19.9	0.080	0.25
Q6	Smoking	19.5	19.5	0.007	0.30
Q48	Preference for health-professional recommendations	19.4	15.0	0.105	0.23
Q56	Willing to pay more for safe, effective products	19.3	11.9	0.105	0.23
Q33	Excessive sun favors cutaneous oxidative stress	18.5	15.0	0.103	0.24
Q18	Food frequency: ultra-processed foods	18.5	14.1	0.178	0.21
Q45	Concern about adverse effects of cosmetics	18.5	14.9	0.155	0.22
Q50	Packaging/marketing influence purchase	18.1	10.8	0.250	0.19
Q10	Perceived stress level	17.7	13.2	0.098	0.24
Q38	SPF and antioxidants offer complementary protection	17.6	15.0	0.178	0.21
Q37	Antioxidant products require constant use	17.4	12.8	0.235	0.20
Q18	Food frequency: citrus	17.1	17.1	0.103	0.23
Q7	Alcohol consumption	16.8	16.8	0.019	0.25
Q24	Antioxidant-rich food consumption	16.7	14.4	0.138	0.22
Q9	Sports activity frequency	16.4	15.4	0.096	0.24
Q34	Antioxidants reduce oxidative-stress effects	15.4	15.4	0.459	0.16
Q18	Food frequency: green vegetables	14.7	10.9	0.453	0.16
Q36	Smoking accelerates oxidative processes	14.7	11.2	0.137	0.22
Q18	Food frequency: nuts/seeds	14.7	11.9	0.422	0.17
Q54	Interest in learning more about antioxidants	14.5	14.5	0.263	0.19
Q18	Food frequency: oily fish	13.9	11.5	0.263	0.19
Q28	Attention to diet for skin health	12.2	12.2	0.604	0.14
Q25	Antioxidant beverage consumption (e.g., green tea)	12.2	11.4	0.459	0.16
Q51	Lifestyle more important than cosmetics	10.6	8.2	0.476	0.15

Q2	Age (groups)	9.9	9.9	0.147	0.20
Q29	Adequate fluid intake for skin health	9.5	9.2	0.750	0.11
Q18	Food frequency: dark chocolate	9.3	5.5	0.851	0.09
Q58	Self-rated skin health	6.6	6.5	0.552	0.14
Q1	Sex	6.5	6.5	0.452	0.07
Q27	Antioxidant supplement frequency	6.1	6.1	0.851	0.09
Q5	Residence	3.8	3.8	0.633	0.04
Q8	Average sleep hours/night	3.3	2.7	0.888	0.06
Q17	Daily fruit/vegetable intake	3.0	3.0	0.750	0.03
Q16	Adverse reactions to antioxidant cosmetics	2.6	2.6	0.663	0.04
Q3	Field of study	0.0	0.0	—	—
Q4	Year of study	0.0	0.0	—	—

Supplementary Table S4. Polychoric-based sensitivity analyses. (a) Exploratory factor analysis pattern matrix estimated from the polychoric correlation matrix of the 36 candidate items (minimum-residual extraction, promax rotation, four factors), with communalities (h2). Loadings of at least 0.40 are shown in bold. Polychoric correlations exceeded their Pearson counterparts by 0.02 on average (maximum absolute difference 0.065); the four-factor solution explained 38.1% of the common variance, and Tucker congruence coefficients between the Pearson-based and polychoric-based factors were 0.998-1.000. (b) Fit of the a priori and refined confirmatory factor analysis models estimated by unweighted least squares on the polychoric correlation matrix. F1, Knowledge; F2, Behavioral Intention; F3, Nutrition/Lifestyle; F4, Attitudes; (R), reverse-scored item.

(a) Polychoric EFA pattern matrix

Item	Domain (a priori)	F1	F2	F3	F4	h2
Q22	Nutrition/Lifestyle	0.01	−0.03	**0.58**	0.09	0.35
Q23	Nutrition/Lifestyle	0.02	0.03	**0.66**	−0.00	0.43
Q24	Nutrition/Lifestyle	0.01	0.05	**0.68**	−0.03	0.47
Q25	Nutrition/Lifestyle	−0.06	−0.01	**0.59**	0.02	0.36
Q26 (R)	Nutrition/Lifestyle	−0.00	−0.03	**0.59**	−0.01	0.35
Q27	Nutrition/Lifestyle	0.04	0.00	0.00	0.16	0.03
Q28	Nutrition/Lifestyle	0.22	−0.01	**0.61**	0.00	0.41
Q29	Nutrition/Lifestyle	−0.01	−0.02	**0.74**	−0.02	0.55
Q30	Nutrition/Lifestyle	−0.00	0.01	**0.64**	−0.04	0.42
Q31	Nutrition/Lifestyle	**0.72**	−0.02	−0.00	0.06	0.52
Q32	Knowledge	**0.71**	0.03	−0.02	0.03	0.51
Q33	Knowledge	**0.70**	0.05	0.01	0.05	0.49
Q34	Knowledge	**0.64**	0.09	−0.01	0.01	0.42
Q35	Knowledge	**0.71**	0.02	0.10	0.06	0.52
Q36	Knowledge	**0.67**	0.04	−0.02	0.01	0.45
Q37	Knowledge	**0.68**	0.03	−0.05	0.12	0.48
Q38	Knowledge	**0.68**	0.01	−0.03	0.07	0.47
Q39	Knowledge	**0.67**	0.07	−0.01	0.01	0.45
Q40	Knowledge	**0.72**	0.01	0.02	0.01	0.51
Q41 (R)	Knowledge	**0.64**	0.02	0.01	−0.05	0.42
Q42	Attitudes	0.06	0.23	0.04	**0.47**	0.28

Q43	Attitudes	0.04	0.23	−0.01	**0.47**	0.27
Q44	Attitudes	0.03	0.25	0.04	**0.43**	0.25
Q45	Attitudes	0.00	−0.03	0.01	0.11	0.01
Q46	Attitudes	−0.04	0.03	0.02	0.04	0.00
Q47	Attitudes	−0.04	0.01	0.00	0.03	0.00
Q48	Attitudes	0.28	0.23	−0.01	**0.40**	0.29
Q49	Attitudes	0.03	−0.08	−0.02	0.15	0.03
Q50	Attitudes	−0.01	0.05	−0.04	0.02	0.00
Q51	Attitudes	**0.61**	0.02	0.13	0.08	0.40
Q52	Intention	0.05	**0.73**	0.05	−0.07	0.54
Q53	Intention	0.01	**0.75**	0.00	−0.06	0.57
Q54	Intention	0.08	**0.76**	0.03	−0.12	0.60
Q55	Intention	0.03	**0.86**	0.03	−0.19	0.77
Q56	Intention	0.06	**0.68**	0.01	−0.09	0.47
Q57	Intention	0.08	**0.77**	−0.03	−0.12	0.62

(b) CFA fit on the polychoric correlation matrix (unweighted least squares)

**Model**	**CFI**	**TLI**	**RMSEA**	**Std. loadings (min)**	**Std. loadings (max)**
A priori (36 items)	0.933	0.928	0.096	−0.01	0.79
Refined (28 items)	0.990	0.990	0.043	0.53	0.78
