# Supplementary material for: Development and Pilot Validation of the Romanian Antioxidant Cosmetics and Nutrition Questionnaire (RO-ACNQ): A Simulation-Based Multicenter Benchmark with an Observed Pilot Sample
Source: Antioxidants (Basel). 2026 Oct 3;15(10):1282. doi: 10.3390/antiox15101282 (PMC13700888; doi:10.3390/antiox15101282)
Supplement: Supplementary file 1 [file antioxidants-15-01282-s001.zip › File S1 Questionnaire Antioxidants_EN+RO.pdf]

Questionnaire on Knowledge, Attitudes, and Usage of Antioxidant Cosmetic Products and Associated Nutritional Behaviors among University Students

Introduction and Participant Consent

Study Invitation: You are invited to participate in a research study assessing antioxidant-related knowledge, attitudes, nutrition/lifestyle behaviors, and the use of and intentions toward antioxidant cosmetic products among university students.

Participation and Procedure: Participation involves completing the RO-ACNQ questionnaire and takes approximately 12–15 minutes. The study is observational and involves no medical intervention. Completing the questionnaire is not expected to involve medical risks and does not replace professional medical advice.

Confidentiality and Data Protection: Responses are collected anonymously, and no names, contact details, or other directly identifying information will be recorded. Data will be stored securely and processed in accordance with the General Data Protection Regulation (GDPR, EU 2016/679). Results will be analyzed and reported only in aggregate form for scientific and academic purposes.

Voluntary Participation: Participation is entirely voluntary. You may discontinue the questionnaire at any time before submission by closing the form, without any consequences. By submitting the completed questionnaire, you consent to the anonymous use of your responses for scientific research, academic analyses, a doctoral dissertation, and scientific publications.

Participant Agreement: By proceeding with and submitting the questionnaire, you confirm that you have read and understood the information above, voluntarily agree to participate in the study, and consent to the anonymous processing and use of your responses for the scientific purposes described.above.

Do you agree to participate in this study? *

- ☐ Yes
- ☐ No

1. Sex: *

- ☐ Female
- ☐ Male

2. Age (years): *

- ☐ ____________________

3. Field of study: *

- ☐ Medicine
- ☐ Pharmacy
- ☐ Nursing / Medical Assistance
- ☐ Nutrition / Dietetics
- ☐ Other: ____________________

4. Year of study: *

- ☐ Year I
- ☐ Year II
- ☐ Year III
- ☐ Year IV
- ☐ Year V
- ☐ Year VI
- ☐ Master's / Ph.D.

5. Area of residence: *

- ☐ Urban
- ☐ Rural

6. Do you smoke? *

☐ Yes
☐ No

7. Do you consume alcohol? *

☐ Never
☐ Rarely
☐ Occasionally
☐ Frequently

8. Average number of sleep hours per night: *

☐ < 5 hours
☐ 5–6 hours
☐ 7–8 hours
☐ > 8 hours

9. How often do you engage in sports/physical activities? *

☐ Never
☐ Rarely
☐ 1–2 times/week
☐ 3–4 times/week
☐ Daily

10. Perceived stress level: *

☐ Very low
☐ Low
☐ Moderate
☐ High
☐ Very high

11. Do you use cosmetic products containing antioxidant ingredients? *

☐ Yes
☐ No

12. Which types of products do you frequently use? *

☐ Creams
☐ Serums
☐ Lotions
☐ Masks
☐ Facial cleansers
☐ Sunscreens / Sun protection products
☐ Body care products
☐ Other products: ______________________

13. Frequency of use: *

☐ Daily
☐ Several times a week
☐ Weekly
☐ Occasionally

14. Who influences your choice of products? *

☐ Dermatologist
☐ Pharmacist
☐ Cosmetic store staff
☐ Family / Friends
☐ Influencers / Social media
☐ Advertisements

☐ I choose independently, based on personal research
☐ Someone else: ____________________

15. What is the primary reason for using antioxidant products? *

☐ Preventing skin aging
☐ Reducing wrinkles
☐ Improving skin appearance
☐ Skin hydration
☐ Reducing spots / hyperpigmentation
☐ Protection against environmental factors
☐ Specialist recommendation
☐ Other reason: ____________________

16. Have you experienced adverse reactions to antioxidant cosmetic products? *

☐ Yes
☐ No

17. Do you consume fruits and vegetables daily? *

☐ Yes
☐ No

18. How frequently do you consume: (1 = Never; 5 = Very frequently) *

- **Berries** ☐ 1 ☐ 2 ☐ 3 ☐ 4 ☐ 5
- **Citrus fruits** ☐ 1 ☐ 2 ☐ 3 ☐ 4 ☐ 5
- **Green leafy vegetables** ☐ 1 ☐ 2 ☐ 3 ☐ 4 ☐ 5
- **Nuts / Seeds** ☐ 1 ☐ 2 ☐ 3 ☐ 4 ☐ 5
- **Green tea** ☐ 1 ☐ 2 ☐ 3 ☐ 4 ☐ 5
- **Dark chocolate** ☐ 1 ☐ 2 ☐ 3 ☐ 4 ☐ 5
- **Fatty fish** ☐ 1 ☐ 2 ☐ 3 ☐ 4 ☐ 5
- **Ultra-processed foods** ☐ 1 ☐ 2 ☐ 3 ☐ 4 ☐ 5
- **Energy drinks** ☐ 1 ☐ 2 ☐ 3 ☐ 4 ☐ 5

19. Do you use antioxidant supplements (vitamins, minerals, etc.)? *

☐ Yes
☐ No

20. If yes: *

☐ Vitamin C
☐ Vitamin E
☐ Zinc
☐ Selenium
☐ Collagen
☐ Omega-3
☐ Other supplements

21. Do you believe diet influences skin health? *

☐ 1 (Not at all)
☐ 2
☐ 3
☐ 4
☐ 5 (To a very great extent)

22. How frequently do you consume: *

a. Fresh fruits

- ☐ Never
- ☐ Rarely
- ☐ Sometimes
- ☐ Frequently
- ☐ Very frequently

b. Fresh vegetables

- ☐ Never
- ☐ Rarely
- ☐ Sometimes
- ☐ Frequently
- ☐ Very frequently

c. Foods rich in antioxidants

- ☐ Never
- ☐ Rarely
- ☐ Sometimes
- ☐ Frequently
- ☐ Very frequently

d. Beverages with antioxidant potential

- ☐ Never
- ☐ Rarely
- ☐ Sometimes
- ☐ Frequently
- ☐ Very frequently

e. Ultra-processed products

- ☐ Never
- ☐ Rarely
- ☐ Sometimes
- ☐ Frequently
- ☐ Very frequently

f. Antioxidant supplements

- ☐ Never
- ☐ Rarely
- ☐ Sometimes
- ☐ Frequently
- ☐ Very frequently

23. How frequently do you: *

a. Pay attention to your diet for skin health

- ☐ Never
- ☐ Rarely
- ☐ Sometimes
- ☐ Frequently
- ☐ Very frequently

b. Consume an adequate amount of water / fluids

- ☐ Never
- ☐ Rarely
- ☐ Sometimes
- ☐ Frequently
- ☐ Very frequently

c. Maintain a balanced diet

- ☐ Never
- ☐ Rarely
- ☐ Sometimes
- ☐ Frequently
- ☐ Very frequently

d. Notice that stress and diet affect your skin

- ☐ Never
- ☐ Rarely
- ☐ Sometimes
- ☐ Frequently
- ☐ Very frequently

24. Please indicate the extent to which you agree with the following statements regarding antioxidants and oxidative stress: *

a. Free radicals can contribute to premature skin aging

- ☐ Strongly disagree
- ☐ Disagree
- ☐ Neither agree nor disagree
- ☐ Agree
- ☐ Strongly agree

b. Excessive sun exposure promotes oxidative stress

- ☐ Strongly disagree
- ☐ Disagree
- ☐ Neither agree nor disagree

- ☐ Agree
- ☐ Strongly agree

- **c. Antioxidants can reduce the harmful effects of pollution and UV radiation**
  - ☐ Strongly disagree
  - ☐ Disagree
  - ☐ Neither agree nor disagree
  - ☐ Agree
  - ☐ Strongly agree

d. A diet rich in fruits and vegetables supports skin health

- ☐ Strongly disagree
- ☐ Disagree
- ☐ Neither agree nor disagree
- ☐ Agree
- ☐ Strongly agree

- **e. Smoking accelerates skin degradation processes through oxidative stress**
  - ☐ Strongly disagree
  - ☐ Disagree
  - ☐ Neither agree nor disagree
  - ☐ Agree
  - ☐ Strongly agree

f. Antioxidant products must be used over a longer period to achieve visible effects

- ☐ Strongly disagree
- ☐ Disagree
- ☐ Neither agree nor disagree
- ☐ Agree
- ☐ Strongly agree

g. SPF and antioxidants can exert a synergistic effect in skin protection

- ☐ Strongly disagree
- ☐ Disagree
- ☐ Neither agree nor disagree
- ☐ Agree
- ☐ Strongly agree

h. Insufficient sleep can impair the skin's capacity to regenerate

- ☐ Strongly disagree
- ☐ Disagree
- ☐ Neither agree nor disagree
- ☐ Agree
- ☐ Strongly agree

i. Pollution can contribute to the impairment of the skin barrier

- ☐ Strongly disagree
- ☐ Disagree
- ☐ Neither agree nor disagree
- ☐ Agree
- ☐ Strongly agree

j. I do not consider myself sufficiently informed about the role of antioxidants

- ☐ Strongly disagree
- ☐ Disagree
- ☐ Neither agree nor disagree
- ☐ Agree

- ☐ Strongly agree

25. Please indicate the extent to which you agree with the following statements regarding antioxidant cosmetic products: *

a. I consider antioxidant products essential in my skincare routine
- ☐ Strongly disagree
- ☐ Disagree
- ☐ Neither agree nor disagree
- ☐ Agree
- ☐ Strongly agree

b. I prefer cosmetic products formulated with natural antioxidant ingredients
- ☐ Strongly disagree
- ☐ Disagree
- ☐ Neither agree nor disagree
- ☐ Agree
- ☐ Strongly agree

c. I trust the efficacy of dermatocosmetic products containing antioxidants
- ☐ Strongly disagree
- ☐ Disagree
- ☐ Neither agree nor disagree
- ☐ Agree
- ☐ Strongly agree

d. I am concerned about the side effects of certain cosmetic products
- ☐ Strongly disagree
- ☐ Disagree
- ☐ Neither agree nor disagree
- ☐ Agree
- ☐ Strongly agree

e. I believe antioxidant products can substitute a healthy lifestyle
- ☐ Strongly disagree
- ☐ Disagree
- ☐ Neither agree nor disagree
- ☐ Agree
- ☐ Strongly agree

f. I consider information regarding antioxidant products to be easily accessible
- ☐ Strongly disagree
- ☐ Disagree
- ☐ Neither agree nor disagree
- ☐ Agree
- ☐ Strongly agree

g. I would rely on recommendations provided by a dermatologist or pharmacist
- ☐ Strongly disagree
- ☐ Disagree
- ☐ Neither agree nor disagree
- ☐ Agree
- ☐ Strongly agree

h. Price influences my selection of antioxidant products
- ☐ Strongly disagree
- ☐ Disagree
- ☐ Neither agree nor disagree

- ☐ Agree
- ☐ Strongly agree

i. Packaging and marketing influence my purchasing decision

- ☐ Strongly disagree
- ☐ Disagree
- ☐ Neither agree nor disagree
- ☐ Agree
- ☐ Strongly agree

j. I believe lifestyle is more important than the cosmetic products used

- ☐ Strongly disagree
- ☐ Disagree
- ☐ Neither agree nor disagree
- ☐ Agree
- ☐ Strongly agree

26. Please indicate the extent to which you agree with the following statements regarding your intention to use antioxidant products: *

a. I intend to use antioxidant products on a long-term basis

- ☐ Strongly disagree
- ☐ Disagree
- ☐ Neither agree nor disagree
- ☐ Agree
- ☐ Strongly agree

b. I would recommend antioxidant products to others

- ☐ Strongly disagree
- ☐ Disagree
- ☐ Neither agree nor disagree
- ☐ Agree
- ☐ Strongly agree

c. I am interested in learning more information about antioxidants

- ☐ Strongly disagree
- ☐ Disagree
- ☐ Neither agree nor disagree
- ☐ Agree
- ☐ Strongly agree

d. I would use antioxidant products if provided with samples or specialist recommendations

- ☐ Strongly disagree
- ☐ Disagree
- ☐ Neither agree nor disagree
- ☐ Agree
- ☐ Strongly agree

e. I am willing to pay more for high-quality antioxidant products

- ☐ Strongly disagree
- ☐ Disagree
- ☐ Neither agree nor disagree
- ☐ Agree
- ☐ Strongly agree

f. I would use antioxidant products even without a recommendation from a specialist

- ☐ Strongly disagree
- ☐ Disagree

- ☐ Neither agree nor disagree
- ☐ Agree
- ☐ Strongly agree

27. How would you rate the overall health of your skin? *

☐ Very poor
☐ Poor
☐ Moderate
☐ Good
☐ Very good

28. In the past 12 months, have you experienced: *

☐ Acne
☐ Oily skin
☐ Xerosis / Dry skin
☐ Cutaneous sensitivity
☐ Hyperpigmentation
☐ Dermatitis
☐ None

29. I consider my skin to be affected by: *

☐ Stress
☐ Diet / Nutrition
☐ Lack of sleep
☐ Pollution
☐ Sun exposure
☐ Cosmetic products
☐ Hormonal factors

30. I consider that my current lifestyle supports skin health *

☐ 1 (Strongly disagree)
☐ 2
☐ 3
☐ 4
☐ 5 (Strongly agree)

Thank you for your participation!

Chestionar privind cunoștințele, atitudinile și utilizarea produselor antioxidante cosmetice și a comportamentelor nutriționale asociate la studenți universitari

Introducere și consimțământul participantului

Invitație de participare la studiu: Sunteți invitat(ă) să participați la un studiu de cercetare care evaluează cunoștințele referitoare la antioxidanți, atitudinile, comportamentele privind alimentația și stilul de viață, precum și utilizarea și intenția de utilizare a produselor cosmetice cu antioxidanți în rândul studenților.

Participare și procedură: Participarea presupune completarea chestionarului RO-ACNQ și durează aproximativ 12–15 minute. Studiul este observațional și nu implică nicio intervenție medicală. Completarea chestionarului nu presupune riscuri medicale anticipate și nu înlocuiește sfatul sau consultația unui profesionist din domeniul sănătății.

Confidențialitate și protecția datelor: Răspunsurile sunt colectate anonim și nu vor fi înregistrate nume, date de contact sau alte informații care permit identificarea directă a participanților. Datele vor fi stocate în condiții de siguranță și prelucrate în conformitate cu Regulamentul general privind protecția datelor (GDPR, Regulamentul UE 2016/679). Rezultatele vor fi analizate și raportate exclusiv în formă agregată, în scopuri științifice și academice.

Participare voluntară: Participarea este în întregime voluntară. Puteți întrerupe completarea chestionarului în orice moment înainte de transmiterea acestuia, prin închiderea formularului, fără nicio consecință. Prin transmiterea chestionarului completat, vă exprimați consimțământul pentru utilizarea anonimă a răspunsurilor dumneavoastră în scopuri de cercetare științifică, analize academice, elaborarea unei teze de doctorat și publicații științifice.

Acordul participantului: Prin continuarea și transmiterea chestionarului, confirmați că ați citit și ați înțeles informațiile de mai sus, că sunteți de acord să participați în mod voluntar la studiu și că vă exprimați consimțământul pentru prelucrarea și utilizarea anonimă a răspunsurilor dumneavoastră în scopurile științifice descrise mai sus.

Întrebarea 1

Sunteți de acord să participați la acest studiu?*

Variante:

- Da
- Nu

1. Sex: *

- ☐ Feminin
- ☐ Masculin

2. Vârsta (ani): * ___________

3. Domeniul de studiu: *

- ☐ Medicină
- ☐ Farmacie
- ☐ Asistență medicală
- ☐ Nutriție / Dietetică
- ☐ Altul: ____________________

4. Anul de studiu: *

- ☐ Anul I
- ☐ Anul II
- ☐ Anul III
- ☐ Anul IV
- ☐ Anul V
- ☐ Anul VI

- ☐ Master / Doctorat

5. Mediul de rezidență: *

- ☐ Urban
- ☐ Rural

6. Fumați? *

- ☐ Da
- ☐ Nu

7. Consumați alcool? *

- ☐ Niciodată
- ☐ Rareori
- ☐ Ocazional
- ☐ Frecvent

8. Numărul mediu de ore de somn/noapte: *

- ☐ < 5 ore
- ☐ 5–6 ore
- ☐ 7–8 ore
- ☐ > 8 ore

9. Cât de des practicați activități sportive? *

- ☐ Niciodată
- ☐ Rareori
- ☐ 1–2 ori/săptămână
- ☐ 3–4 ori/săptămână
- ☐ Zilnic

10. Nivelul perceput de stres *

- ☐ Foarte scăzut
- ☐ Scăzut
- ☐ Moderat
- ☐ Ridicat
- ☐ Foarte ridicat

11. Utilizați produse cosmetice cu ingrediente antioxidante? *

- ☐ Da
- ☐ Nu

12. Ce tipuri de produse utilizați frecvent? *

- ☐ Creme
- ☐ Seruri
- ☐ Loțiuni
- ☐ Măști
- ☐ Produse pentru curățarea feței
- ☐ Produse pentru protecție solară
- ☐ Produse pentru îngrijirea corpului
- ☐ Alte produse: ____________________

13. Frecvența utilizării: *

- ☐ Zilnic
- ☐ De câteva ori pe săptămână
- ☐ Săptămânal
- ☐ Ocazional

14. Cine influențează alegerea produselor? *

- ☐ Dermatologul
- ☐ Farmacistul

- ☐ Personalul din magazinele de cosmetice
- ☐ Familia/prietenii
- ☐ Influenceri / social media
- ☐ Reclamele
- ☐ Aleg singur(ă), pe baza informațiilor găsite
- ☐ Altcineva: ____________________

15. Care este principalul motiv pentru utilizarea produselor antioxidante? *

- ☐ Prevenirea îmbătrânirii pielii
- ☐ Reducerea ridurilor
- ☐ Îmbunătățirea aspectului pielii
- ☐ Hidratarea pielii
- ☐ Reducerea petelor/pigmentării
- ☐ Protecția împotriva factorilor de mediu
- ☐ Recomandarea unui specialist
- ☐ Alt motiv: ____________________

16. Ați prezentat reacții adverse la produse cosmetice antioxidante? *

- ☐ Da
- ☐ Nu

17. Consumați zilnic fructe și legume? *

- ☐ Da
- ☐ Nu

18. Consumați frecvent: (1 = niciodată; 5 = foarte frecvent) *

- **Fructe de pădure** ☐ 1 ☐ 2 ☐ 3 ☐ 4 ☐ 5
- **Citrice** ☐ 1 ☐ 2 ☐ 3 ☐ 4 ☐ 5
- **Legume verzi** ☐ 1 ☐ 2 ☐ 3 ☐ 4 ☐ 5
- **Nuci/semințe** ☐ 1 ☐ 2 ☐ 3 ☐ 4 ☐ 5
- **Ceai verde** ☐ 1 ☐ 2 ☐ 3 ☐ 4 ☐ 5
- **Ciocolată neagră** ☐ 1 ☐ 2 ☐ 3 ☐ 4 ☐ 5
- **Pește gras** ☐ 1 ☐ 2 ☐ 3 ☐ 4 ☐ 5
- **Produse ultraprocesate** ☐ 1 ☐ 2 ☐ 3 ☐ 4 ☐ 5
- **Bauturi energizante** ☐ 1 ☐ 2 ☐ 3 ☐ 4 ☐ 5

19. Utilizați suplimente antioxidante(vitamine, minerale, etc)? *

- ☐ Da
- ☐ Nu

20. Dacă da: *

- ☐ Vitamina C
- ☐ Vitamina E
- ☐ Zinc
- ☐ Seleniu
- ☐ Colagen
- ☐ Omega-3
- ☐ Alte suplimente

21. Considerați că alimentația influențează sănătatea pielii? *

- ☐ 1 (Deloc)
- ☐ 2
- ☐ 3
- ☐ 4
- ☐ 5 (În foarte mare măsură)

22. Cât de des consumați: *
a. fructe proaspete
- ☐ Niciodată
- ☐ Rar
- ☐ Uneori
- ☐ Frecvent
- ☐ Foarte frecvent

b. legume proaspete
- ☐ Niciodată
- ☐ Rar
- ☐ Uneori
- ☐ Frecvent
- ☐ Foarte frecvent

c. alimente bogate în antioxidanți
- ☐ Niciodată
- ☐ Rar
- ☐ Uneori
- ☐ Frecvent
- ☐ Foarte frecvent

d. băuturi cu potențial antioxidant
- ☐ Niciodată
- ☐ Rar
- ☐ Uneori
- ☐ Frecvent
- ☐ Foarte frecvent

e. produse ultraprocesate
- ☐ Niciodată
- ☐ Rar
- ☐ Uneori
- ☐ Frecvent
- ☐ Foarte frecvent

f. suplimente antioxidante
- ☐ Niciodată
- ☐ Rar
- ☐ Uneori
- ☐ Frecvent
- ☐ Foarte frecvent

23. Cât de frecvent: *
a. acordați atenție alimentației pentru sănătatea pielii
- ☐ Niciodată
- ☐ Rar
- ☐ Uneori
- ☐ Frecvent
- ☐ Foarte frecvent

b. consumați o cantitate adecvată de apă / lichide
- ☐ Niciodată
- ☐ Rar
- ☐ Uneori
- ☐ Frecvent

- ☐ Foarte frecvent

c. aveți o alimentație echilibrată

- ☐ Niciodată
- ☐ Rar
- ☐ Uneori
- ☐ Frecvent
- ☐ Foarte frecvent

d. observați că stresul și alimentația vă afectează pielea

- ☐ Niciodată
- ☐ Rar
- ☐ Uneori
- ☐ Frecvent
- ☐ Foarte frecvent

24. Vă rugăm să indicați în ce măsură sunteți de acord cu următoarele afirmații privind antioxidanții și stresul oxidativ: *

a. Radicalii liberi pot contribui la îmbătrânirea prematură a pielii

- ☐ Dezacord total
- ☐ Dezacord
- ☐ Nici acord, nici dezacord
- ☐ Acord
- ☐ Acord total

b. Expunerea excesivă la soare favorizează stresul oxidativ

- ☐ Dezacord total
- ☐ Dezacord
- ☐ Nici acord, nici dezacord
- ☐ Acord
- ☐ Acord total

c. Antioxidanții pot reduce efectele nocive ale poluării și ale radiațiilor UV

- ☐ Dezacord total
- ☐ Dezacord
- ☐ Nici acord, nici dezacord
- ☐ Acord
- ☐ Acord total

d. Alimentația bogată în fructe și legume susține sănătatea pielii

- ☐ Dezacord total
- ☐ Dezacord
- ☐ Nici acord, nici dezacord
- ☐ Acord
- ☐ Acord total

e. Fumatul accelerează procesele de degradare a pielii prin stres oxidativ

- ☐ Dezacord total
- ☐ Dezacord
- ☐ Nici acord, nici dezacord
- ☐ Acord
- ☐ Acord total

f. Produsele antioxidante trebuie utilizate pe o perioadă mai lungă pentru a avea efecte vizibile

- ☐ Dezacord total
- ☐ Dezacord
- ☐ Nici acord, nici dezacord

- ☐ Acord
- ☐ Acord total

g. SPF-ul și antioxidanții pot avea un efect sinergic în protecția pielii

- ☐ Dezacord total
- ☐ Dezacord
- ☐ Nici acord, nici dezacord
- ☐ Acord
- ☐ Acord total

h. Somnul insuficient poate afecta capacitatea pielii de a se regenera

- ☐ Dezacord total
- ☐ Dezacord
- ☐ Nici acord, nici dezacord
- ☐ Acord
- ☐ Acord total

i. Poluarea poate contribui la deteriorarea barierei cutanate

- ☐ Dezacord total
- ☐ Dezacord
- ☐ Nici acord, nici dezacord
- ☐ Acord
- ☐ Acord total

j. Nu mă consider suficient informat(ă) despre rolul antioxidanților

- ☐ Dezacord total
- ☐ Dezacord
- ☐ Nici acord, nici dezacord
- ☐ Acord
- ☐ Acord total

25. Vă rugăm să indicați în ce măsură sunteți de acord cu următoarele afirmații privind produsele cosmetice antioxidante: *

a. Consider produsele antioxidante esențiale în rutina mea de îngrijire

- ☐ Dezacord total
- ☐ Dezacord
- ☐ Nici acord, nici dezacord
- ☐ Acord
- ☐ Acord total

b. Prefer produsele cosmetice cu ingrediente naturale antioxidante

- ☐ Dezacord total
- ☐ Dezacord
- ☐ Nici acord, nici dezacord
- ☐ Acord
- ☐ Acord total

c. Am încredere în eficiența produselor dermatocosmetice ce conțin antioxidanți

- ☐ Dezacord total
- ☐ Dezacord
- ☐ Nici acord, nici dezacord
- ☐ Acord
- ☐ Acord total

d. Sunt preocupat(ă) de efectele secundare ale unor produse cosmetice

- ☐ Dezacord total
- ☐ Dezacord

- ☐ Nici acord, nici dezacord
- ☐ Acord
- ☐ Acord total

e. Cred că produsele antioxidante pot înlocui un stil de viață sănătos

- ☐ Dezacord total
- ☐ Dezacord
- ☐ Nici acord, nici dezacord
- ☐ Acord
- ☐ Acord total

f. Consider că informațiile despre produsele antioxidante sunt ușor accesibile

- ☐ Dezacord total
- ☐ Dezacord
- ☐ Nici acord, nici dezacord
- ☐ Acord
- ☐ Acord total

g. Aș mege pe recomandări venite din partea dermatologului sau farmacistului

- ☐ Dezacord total
- ☐ Dezacord
- ☐ Nici acord, nici dezacord
- ☐ Acord
- ☐ Acord total

h. Prețul influențează alegerea produselor antioxidante pe care le cumpăr

- ☐ Dezacord total
- ☐ Dezacord
- ☐ Nici acord, nici dezacord
- ☐ Acord
- ☐ Acord total

i. Ambalajul și marketingul influențează decizia mea de cumpărare

- ☐ Dezacord total
- ☐ Dezacord
- ☐ Nici acord, nici dezacord
- ☐ Acord
- ☐ Acord total

j. Cred că stilul de viață este mai important decât produsele cosmetice folosite

- ☐ Dezacord total
- ☐ Dezacord
- ☐ Nici acord, nici dezacord
- ☐ Acord
- ☐ Acord total

26. Vă rugăm să indicați în ce măsură sunteți de acord cu următoarele afirmații privind intenția dumneavoastră de utilizare a produselor antioxidante: *

a. Intenționez să utilizez produse antioxidante pe termen lung

- ☐ Dezacord total
- ☐ Dezacord
- ☐ Nici acord, nici dezacord
- ☐ Acord
- ☐ Acord total

b. Aș recomanda produse antioxidante și altor persoane

- ☐ Dezacord total
- ☐ Dezacord
- ☐ Nici acord, nici dezacord
- ☐ Acord
- ☐ Acord total

c. Sunt interesat(ă) să aflu mai multe informații despre antioxidanți

- ☐ Dezacord total
- ☐ Dezacord
- ☐ Nici acord, nici dezacord
- ☐ Acord
- ☐ Acord total

d. Aș utiliza produse antioxidante dacă aș primi mostre sau recomandări de la specialiști

- ☐ Dezacord total
- ☐ Dezacord
- ☐ Nici acord, nici dezacord
- ☐ Acord
- ☐ Acord total

e. Sunt dispus(ă) să plătesc mai mult pentru produse antioxidante de calitate

- ☐ Dezacord total
- ☐ Dezacord
- ☐ Nici acord, nici dezacord
- ☐ Acord
- ☐ Acord total

f. Aș utiliza produse antioxidante chiar și fără o recomandare din partea unui specialist

- ☐ Dezacord total
- ☐ Dezacord
- ☐ Nici acord, nici dezacord
- ☐ Acord
- ☐ Acord total

27. Cum apreciați sănătatea generală a pielii dumneavoastră? *

- ☐ Foarte slabă
- ☐ Slabă
- ☐ Moderată
- ☐ Bună
- ☐ Foarte bună

28. Ați avut în ultimele 12 luni: *

- ☐ Acnee
- ☐ Ten gras
- ☐ Xerodermie
- ☐ Sensibilitate cutanată
- ☐ Hiperpigmentare
- ☐ Dermatită
- ☐ Niciuna

29. Consider că pielea mea este afectată de: *

- ☐ Stres
- ☐ Alimentație
- ☐ Lipsa somnului
- ☐ Poluare
- ☐ Expunere solară

- ☐ Produse cosmetice
- ☐ Factori hormonali

30. Consider că stilul meu actual de viață susține sănătatea pielii *

- ☐ 1 (Dezacord total)
- ☐ 2
- ☐ 3
- ☐ 4
- ☐ 5 (Acord total)

Vă mulțumim pentru participare!
